# Updating the genomic taxonomy and epidemiology of *Campylobacter hyointestinalis*

**DOI:** 10.1038/s41598-018-20889-x

**Published:** 2018-02-05

**Authors:** David A. Wilkinson, Andrew J. O’Donnell, Rukhshana N. Akhter, Ahmed Fayaz, Hamish J. Mack, Lynn E. Rogers, Patrick J. Biggs, Nigel P. French, Anne C. Midwinter

**Affiliations:** 1 Molecular Epidemiology and Public Health Laboratory (mEpiLab), Infectious Disease Research Centre, School of Veterinary Science, Massey University, Palmerston North, New Zealand; 2 Faculty of Veterinary Science, University of Liverpool, Leahurst, Cheshire CH64 7TE. United Kingdom; 3 New Zealand Food Safety Science and Research Centre, Massey University, Palmerston North, New Zealand

## Abstract

*Campylobacter hyointestinalis* is a member of an emerging group of zoonotic *Campylobacter* spp. that are increasingly identified in both gastric and non-gastric disease in humans. Here, we discovered *C. hyointestinalis* in three separate classes of New Zealand ruminant livestock; cattle, sheep and deer. To investigate the relevance of these findings we performed a systematic literature review on global *C. hyointestinalis* epidemiology and used comparative genomics to better understand and classify members of the species. We found that *C. hyointestinalis* subspecies *hyointestinalis* has an open pangenome, with accessory gene contents involved in many essential processes such as metabolism, virulence and defence. We observed that horizontal gene transfer is likely to have played an overwhelming role in species diversification, favouring a public-goods-like mechanism of gene ‘acquisition and resampling’ over a tree-of-life-like vertical inheritance model of evolution. As a result, simplistic gene-based inferences of taxonomy by similarity are likely to be misleading. Such genomic plasticity will also mean that local evolutionary histories likely influence key species characteristics, such as host-association and virulence. This may help explain geographical differences in reported *C. hyointestinalis* epidemiology and limits what characteristics may be generalised, requiring further genomic studies of *C. hyointestinalis* in areas where it causes disease.

## Introduction

*Campylobacter* is one of the main causative agents of human bacterial gastroenteritis worldwide^1^. Currently divided into 28 distinct species (http://www.bacterio.net/campylobacter.html)^2,3^, this bacterial genus occupies a variety of different niches, primarily the gastrointestinal tracts of many animal species where it may be either commensal or pathogenic^4^. The most frequently attributed sources of human campylobacteriosis are food (particularly poultry), water, and raw milk^5,6^. Human campylobacteriosis has symptoms including diarrhoea (often bloody), abdominal pain, nausea, and headaches^7^ and is most commonly caused by the species *Campylobacter jejuni* and *Campylobacter coli*^8,9^. However, campylobacteriosis has also been reported to be caused by so-called ‘emerging *Campylobacter* spp.^10^, including *Campylobacter concisus*^11^, *Campylobacter sputorum*^12^, *Campylobacter upsaliensis*^12^, *Campylobacter ureolyticus*^13^ and *Campylobacter hyointestinalis* (see below). *Campylobacter* spp., including the ‘emerging species’, have also been associated with other pathological presentations in humans, including bacteraemia^14–18^, abscesses^19–21^, Crohn’s disease^22,23^, meningitis^24^, neonatal infection and abortion^25^. Improved diagnostic technologies have revolutionised our understanding of the clinical importance of “emerging *Campylobacter* spp.”, while the exponentially increasing availability of genetic and genomic data means that our appreciation of each species’ characteristics and ecological associations is constantly evolving.

The taxonomic classification of *Campylobacter* species has historically been driven by phenotypic characterisation of bacterial isolates, with molecular characterization being only a minor component of the bacterial description^26^. As with all branches of taxonomy this is changing with the advent of widely available and accessible DNA sequencing technologies, but it is only very recently that the standards for description of *Campylobacter* species have been up-dated to a biphasic approach with genotype and phenotype descriptions both being important^27^. DNA-based classifications also have no universal standard, and different methodologies (eg. multi-locus sequence typing vs. whole genome sequencing) allow different resolving capacities for the distinction between strain variants as well as dealing with complex evolutionary phenomena such as recombination in different ways. Nonetheless, taxonomic updates based on DNA sequences are essential and have led to the inclusion of species previously not classified as *Campylobacter*^28^ and the exclusion and reclassification of others^29^.

*Campylobacter hyointestinalis*, originally isolated from diseased pigs^30^ is a member of the “emerging *Campylobacter* spp.” group that can also cause disease in humans. Although *C. hyointestinalis* was originally described as being able to grow at 43 °C^31,32^ it is frequently referred to as a non-thermotolerant species^33^. The species has two recognised subspecies; *hyointestinalis* and *lawsonii*. Molecular detection systems have been published which provide culture independent detection methods for this species^34,35^. The first complete genome sequences of *C. hyointestinalis* subspecies *hyointestinalis* and *lawsonii* were published in 2016^36^, showing that the *C. hyointestinalis* genome was approximately 1.75 Mbp in length, with a GC content of approximately 34%.

Here, we provide an up to date summary of all publicly reported detections of *C. hyointestinalis* infection. We use genomic data generated from New Zealand isolates to examine the evolutionary processes occurring in *C. hyointestinalis*, and discuss how these influence our interpretation of both its characteristics and classification.

## Results

### Literature Survey

We conducted a systematic literature review looking for articles that reported original observations of *C. hyointestinalis*. A total of 75 articles were identified that satisfied the search criteria (inclusion of the text “*Campylobacter hyointestinalis*” at any point) from more than 1,500 articles, using a combination of different search engines for scientific literature. The summary of the data extracted from these 75 articles is provided in Supplementary Data S1.

**Figure 1:**
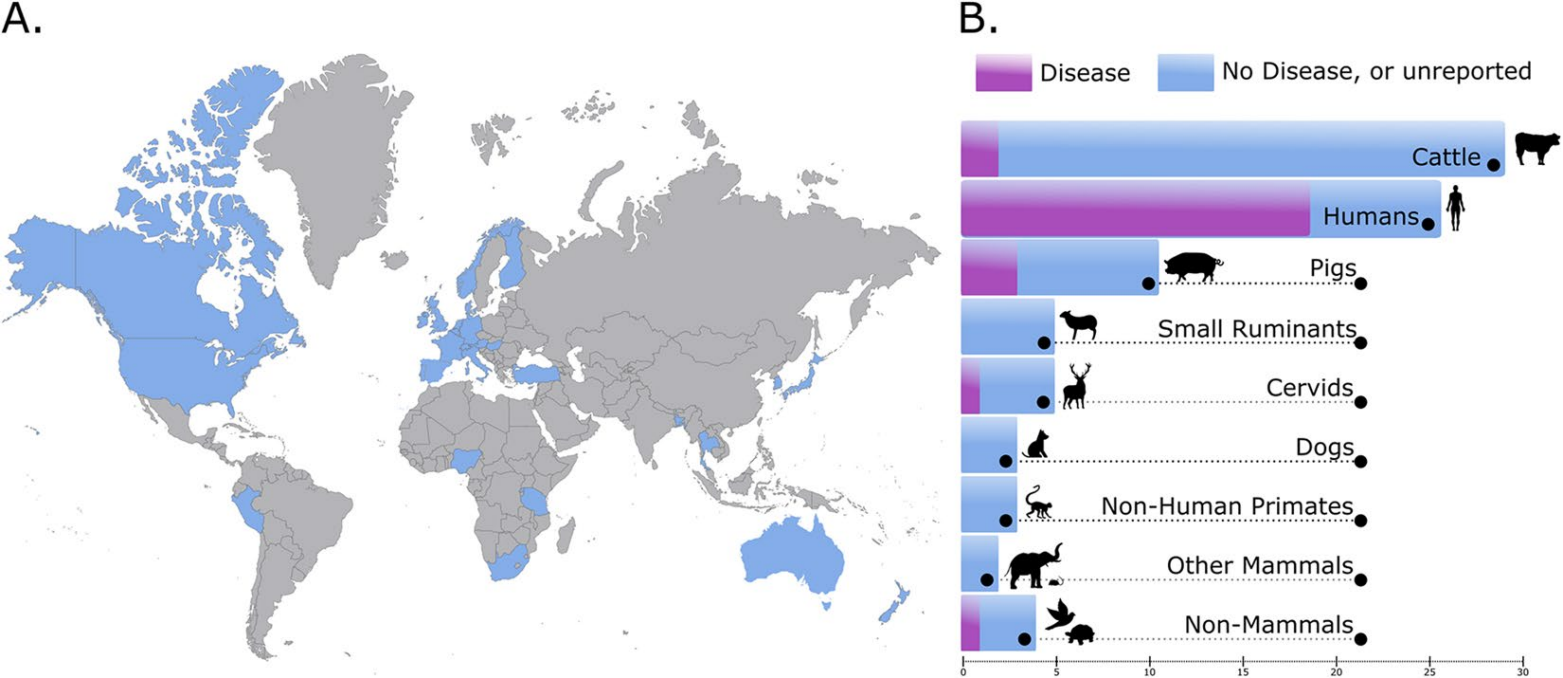
Summary of data from the systematic literature review carried out as part of this study. (**A**) Geographical distribution of publications reporting original observations of *C. hyointestinalis*. Countries that have reported *C. hyointestinalis* are displayed in blue. (**B**) Number of reports of *C. hyointestinalis* from different sources. Bars represent the number of publications that report the presence of *C. hyointestinalis* in each species. Purple bars represent cases where infection was in a host displaying gastric disease, blue bars represent instances where there was no disease, or the disease status of the tested host was not reported. The presented map was modified in Inkscape 0.91 (http://inkscape.org) from World with Countries – Outline by FreeVectorMaps.com. Icons made by Freepik from www.flaticon.com (https://www.flaticon.com/authors/freepik/).

To date, *C. hyointestinalis* has been reported from 30 countries across the world, and from six of the seven continents (Fig. 1A). Although the original reports of *C. hyointestinalis* were from pigs that displayed signs of gastric disease^30^, the most frequently reported source for *C. hyointestinalis* isolation has been the faeces of healthy cattle herds, and humans with various presentations of gastroenteritis (Fig. 1B). Ninety-three percent of all reports of *C. hyointestinalis* isolation have been from mammalian hosts, of which almost all were domesticated or zoo-kept animals. However, select observations of *C. hyointestinalis* have been made from the excretions of non-mammalian hosts, including birds, tortoises and shellfish.

**Figure 2:**
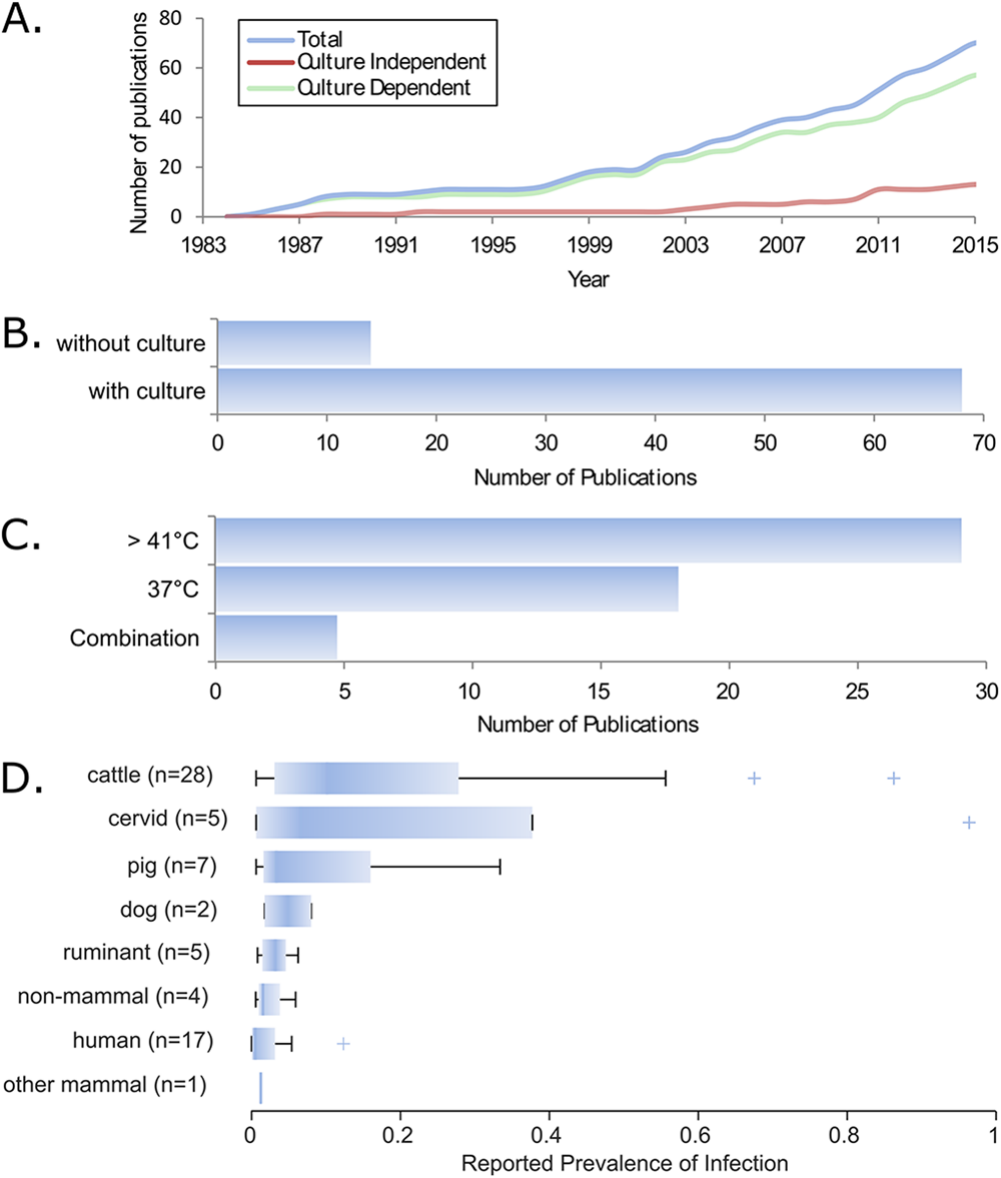
Summary of data from the literature survey carried out as part of this study. (**A**) Temporal trend of publications reporting original observations of *C. hyointestinalis*. (**B**) Number of publications that used culture dependent, or culture independent methods to identify *C. hyointestinalis*. (**C**) Number of publications reporting culture dependent methods with culture conditions at high (>41 °C), or low (37) temperatures for the isolation of *C. hyointestinalis*. (**D**) For population level studies, defined as those with epidemiological sampling protocols and sample sizes greater than 20 individuals, reported infection prevalence is summarised for each host species. Box plots demark 5^th^ and 95^th^ percentiles, interquartile ranges, median and outlying values.

Reports of *C. hyointestinalis* have increased slowly since its description in 1983 (Fig. 2A), suggesting that new technologies have either not been widely adopted or have not significantly improved the isolation and detection this species. The methodologies that have led to *C. hyointestinalis* isolation have remained relatively unchanged in this time, and culture-dependent methodologies remain the most common way of identifying *C. hyointestinalis* (Fig. 2B), despite the introduction of numerous different molecular techniques for the detection of *Campylobacter* over the same time period^34,35,37^. Although *C. hyointestinalis* is considered to be a non-thermotolerant member of the Campylobacteraeceae family^33^, it has been isolated most commonly under selective conditions that exploit high temperatures (>41 °C) (Fig. 2C), possibly because these are the prevailing conditions used for the identification of *Campylobacter* infections which are more commonly associated with the species *C. jejuni* and *C. coli*^38^.

From the original 75 articles, 68 datasets (including some articles describing multiple datasets) were identified reporting *C. hyointestinalis* with population-level sampling strategies and sample sizes greater than 20 test individuals. In these studies, the reported prevalence of infection has been greatest in cattle, cervids (deer and reindeer) and pigs (Fig. 2D).

### Detection of *C. hyointestinalis* in New Zealand Ruminants

*Campylobacter* isolation was performed on faecal samples from 33 dairy, sheep and beef and deer farms across the Manawatu district of the North Island of New Zealand. In total, *Campylobacter* spp. were identified in 52% of ruminant faecal samples tested. *C. jejuni* and/or *C. coli* were identified in 39% of samples, whereas *C. hyointestinalis* was identified in 21% of samples. The use of pooled samples and a second culture condition for cattle and sheep samples is likely to have increased the detection rate of *C. hyointestinalis* in these samples relative to the deer population.

For deer; of 80 faecal samples taken, nine showed *Campylobacter*-like growth on an mCCDA plate (two stag samples, seven hind samples). Two colonies were taken from each for further examination giving a total of 22 isolates for PCR. Sixteen of the isolates and nine red deer were positive for *Campylobacter*, two positives from stags and seven from hinds. Two isolates from a stag tested positive for *C. jejuni* and all tested negative for *C. coli*. More extensive speciation revealed 14 isolates to be positive for *C. hyointestinalis* (seven hinds, one stag).

For cattle; *Campylobacter* was isolated from 94% of pooled faecal samples. PCR identified 57 *C. jejuni* and *C. coli* isolates, and 28 *C. hyointestinalis* isolates. For sheep; *Campylobacter* was isolated from 51% of pooled faecal samples, of which 29 were identified as *C. jejuni* and *C. coli*, and six were identified as *C. hyointestinalis*. Epidemiological findings are summarised in Supplementary Table S1.

From the 48 isolates identified as *C. hyointestinalis*, a subset of 16 was randomly selected to be characterised via whole genome sequencing. Illumina HiSeq 2 × 250 bp sequencing provided good quality draft genome assemblies for all isolates (Supplementary Table S2).

### Population genetics, genomics and evolutionary analysis

**Figure 3:**
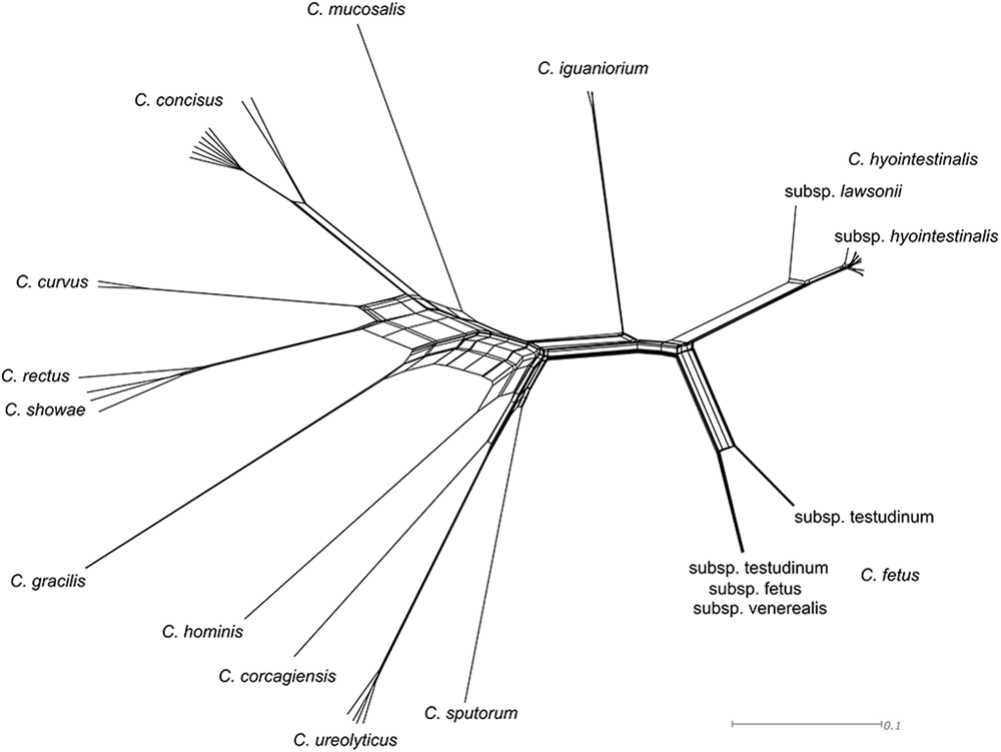
Core genome alignment network for non-thermophilic *Campylobacter* species. Single nucleotide polymorphism distances are calculated from 255 core genes identified by the Genome Profiler software package.

Whole genome MLST analysis identified a conservative total of 255 polymorphic loci that were shared between the 58 genomes included from the non-thermophilic branch of the *Campylobacteraceae*. An annotated list of the 255 conserved, polymorphic loci has been provided in Supplementary Data S2. The resulting network analysis of the concatenated gene alignment from all shared loci had a tree-like structure with clade groupings that essentially matched current species-level taxonomic classifications (Fig. 3). One exception to this was *C. rectus*, which was predicted to have strong similarity to *C. showae*. Those taxa that contained distinct sub-species level taxonomic divisions included *C. hyointestinalis*, *C. fetus* and *C. concisus*. *C. concisus* currently has no formally recognised subspecies, though there are known genomospecies. Conversely, available *C. fetus* data originating from isolates with the subspecies classifications *testudinum*, *fetus* and *venerealis* formed a single distinct branch of the similarity network. *C. hyointestinalis* subsp. *lawsonii* and *hyointestinalis* were clearly distinguishable. All isolates obtained as part of this study belonged to the taxon *C. hyointestinalis* subsp. *hyointestinalis*.

**Figure 4:**
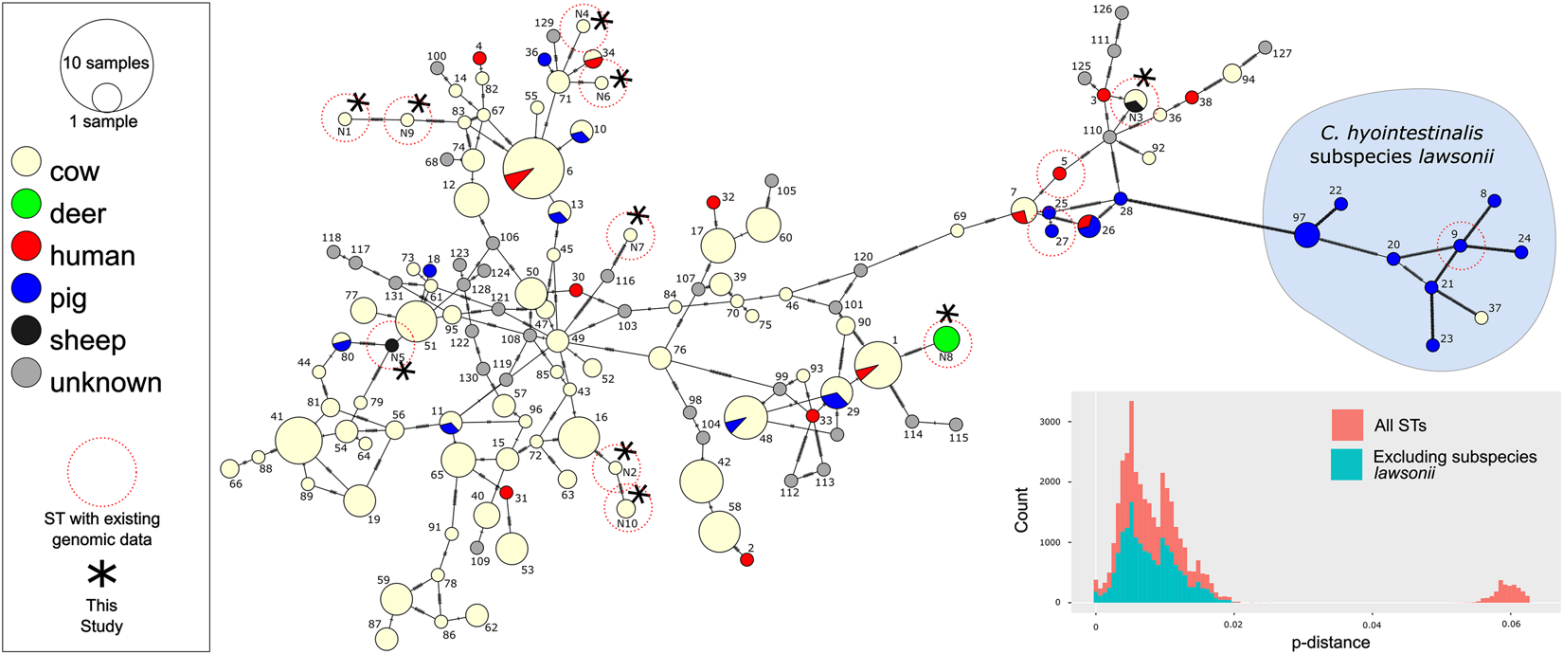
Minimum Spanning Tree of the seven-gene MLST data from *C. hyointestinalis* sourced from PubMLST. Circle diameter signifies the number of reported samples in the PubMLST database, and colour depicts host species as indicated in the legend. Inset, histograms of pairwise p-distance calculations between all samples including (light red) and excluding (light blue) subspecies *lawsonii* from the calculations. Sequence types for which no isolate metadata were available are represented as singletons of unknown source.

Isolates were compared to publicly deposited data from PubMLST by concatenating and aligning the appropriate seven MLST loci from the assembled genomic data. Previously unreported allelic sequences were obtained from many of the isolates, and for all loci except *gltA* and *glyA*. These were used to assign ten new sequence-type definitions (Supplementary Table S3, Supplementary Data S3). Sequence type similarity was calculated for all known *C. hyointestinalis* isolates using the minimum-spanning method (Fig. 4). In this analysis, most New Zealand isolates were located at terminal positions of the network, suggesting significant divergence from *C. hyointestinalis* variants from other geographical locations. However, p-distance analysis confirmed that all of these variants belonged to the same subspecies. Source data from the 237 available isolates and 141 different sequence types analysed showed that 94% of *C. hyointestinalis* subsp. *lawsonii* isolates had originated from swine. Contrastingly, only 6% of *C. hyointestinalis* subsp. *hyointestinalis* were isolated from swine, whereas 89% were isolated from cattle.

We further examined the evolutionary characteristics of New Zealand *C. hyointestinalis* within the seven-gene MLST data by looking for signs of recombination^39^. By this method only 344 bp remained after stripping predicted recombinant sites from the original 3,312 bp alignment, suggesting that approximately 90% of sites within the standard MLST scheme loci may have been subject to recombination at some point (Supplementary Figure 1). Phylogenetic reconstruction from the raw data showed poor node-support, most likely as a result of this estimated high rate of recombination (data not shown). Phylogenetic reconstruction based on the recombination-stripped data had high posterior probabilities for the majority of structure-defining tree nodes, and predicted highly contrasting relationships between members of this species relative to the minimum spanning method (Supplementary Figure 2). Within data derived from our samples, the nature of this difference was most evident for sequence types N1 and N9 (all isolates from New Zealand cattle), which were erroneously assigned to a cluster with international isolates of STs 83, 67 and 6 using the minimum spanning method (Fig. 4) and predicted to have a much closer relationship to ST N5 (an isolate from New Zealand sheep) in the phylogenetic analysis (Supplementary Figure 2).

We extended our analysis of recombination rates to the whole-genome scale. Similar to regions within the seven gene MLST allele sites, recombination events were predicted to be extremely frequent between lineages, with as few as 54,000 bp (4.3%) of the total core genomic content being retained in the global clonal frame. On average, 79% ± SD 9% of genomic content on tree leaves was estimated to have been inherited vertically from its closest estimated ancestor. For isolate S1499c, shared recombination loci between genome sequences of this atypical *C. hyointestinalis* subspecies *hyointestinalis* and *C. hyointestinalis* subsp. *lawsonii* suggested that this isolate had undergone subspecies-level introgression at some point in its evolutionary history (Supplementary Figure 3).

**Figure 5:**
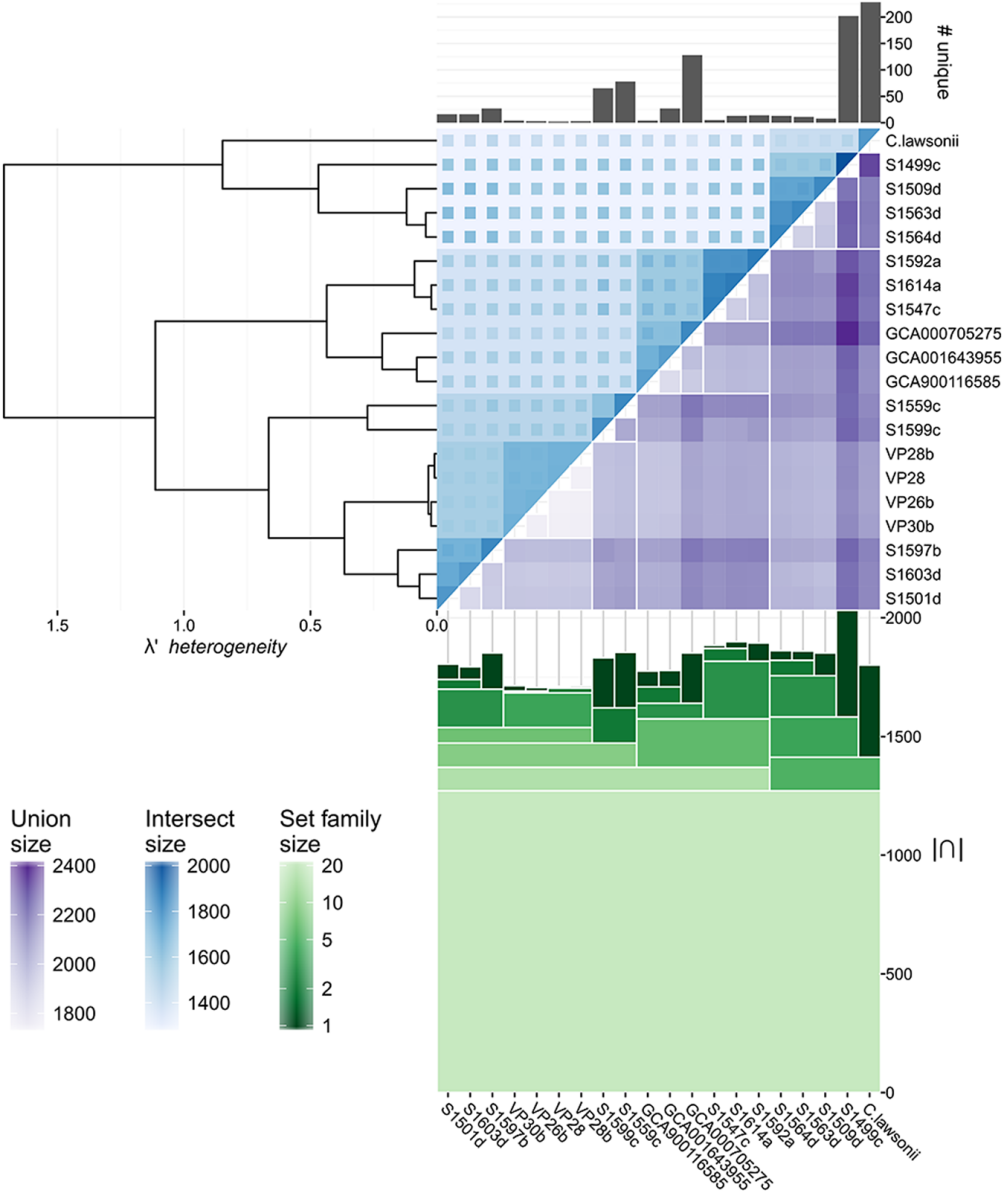
Pangenome structure of *Campylobacter hyointestinalis*. The dendrogram (left) depicts the optimal hierarchical clustering of the orthologous gene group presence/absence matrix generated, expressed in terms of λ′ heterogeneity as described in^72^. The heatmap (centre) depicts pairwise pangenome intersect and union sizes for all genome combinations. Numbers of unique genes associated with each genome are indicated by bars (top). Numbers of gene clusters associated with the depicted genome groups are shown as a stacked icicle plot where the intersect size (number of shared genes) in each set determines the height of each box (bottom). The figure was generated using Pedersen’s HierarchicalSets package for R^72^.

**Figure 6:**
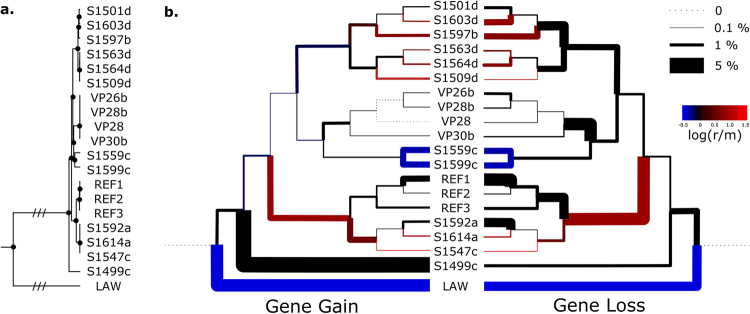
Predicted gene gain, gene loss and recombination/mutation rates with the predicted evolutionary history of *C. hyointestinalis*. (**a**) Recombination-stripped core-gene based phylogeny, generated using BEAST2. (**b**) The same phylogeny as in (**a**), represented as two mirrored cladograms. Branch thickness denotes predicted gene gain (left) and gene loss (right) events as calculated using the Count software package^74^, and expressed as a percentage of the total genomic content. Branch colour denotes the log value of the predicted recombination to mutation ratio for each corresponding branch. Reference genome sequences not generated as part of this study are referred to as REF1 (GCA000705275), REF2 (GCA001643955), REF3 (GCA900116585) and LAW (*C. hyointestinalis* subsp. *lawsonii*, GCA001643975).

**Figure 7:**
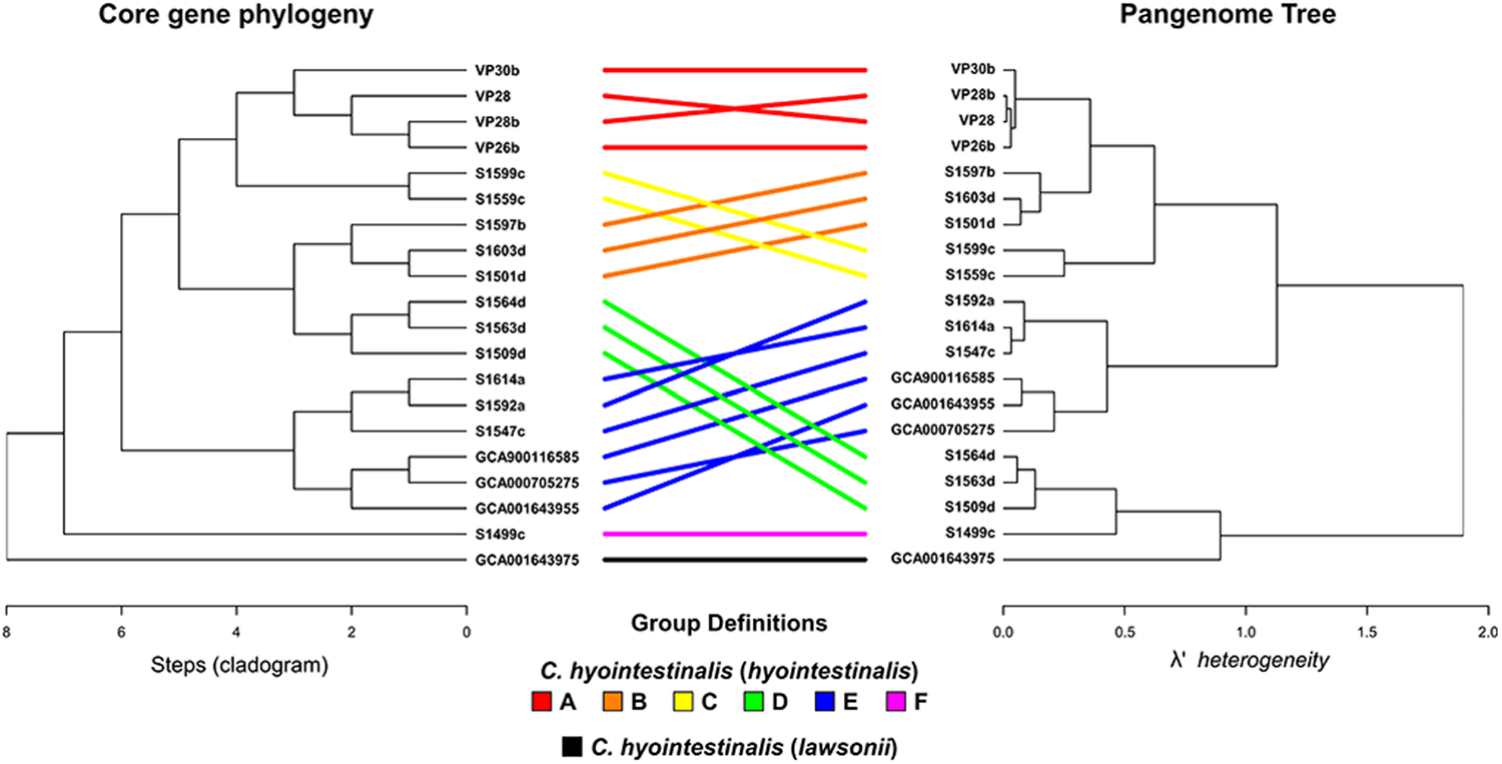
Tanglegram depicting the relationship between the predicted evolutionary histories of *C. hyointestinalis* based on core gene phylogenies (left) and hierarchical clustering of the pangenome presence/absence matrix (right). Correspondences were used to designate clade groups, which are linked in the tanglegram using individual colours per clade.

We next examined core- and pan- genome characteristics for all *C. hyointestinalis* genomes available from our study and both the NCBI and ENA databases. Prokka-based annotation predicted an average of 1,828 coding sequences (±SD 80) for an average genome size of 1.77 Mbp (±SD 0.06 Mbp). No plasmids were detected when queried against the PlasmidFinder database^40^. OrthoMCL-based clustering executed in get_homologues estimated a core genome size of 1,271 genes (67% ± SD 3% of the total number of genes), a soft-core size (defined as orthologous genes found in all or all but one of the studied genomes) of 1,416 genes (78% ± SD 3% of the total number of genes) and a pangenome size of 3,446 genes (Fig. 5). Rarefaction analysis suggested that even when restricted to the subspecies scale, the pangenome of *C. hyointestinalis* could be considered as open, with a Heap’s law coefficient of 0.21 (Supplementary Figure S4). We quantified the rates of gene acquisition and loss along the predicted recombination-stripped phylogeny, using the Count software package and the pan-genome presence/absence matrix generated by get_homologues. Our analysis supported the theory that gene acquisition may drive microbial speciation, with major gene acquisition events being associated with the subspecies level divergence event of *C. hyointesinalis* subsp. *lawsonii*, as well as the atypical isolate S1499c (Fig. 6). Conversely, gene loss events were predicted to occur at more evenly-spaced intervals along the *C. hyointestinalis* evolutionary history. Interestingly, recombination/mutation rates also varied greatly between different *C. hyointestinalis* lineages (Fig. 7) and higher rates of recombination showed no correlation to higher rates of gene gain, suggesting that homologous and non-homologous recombination frequencies are determined by independent mechanisms.

**Figure 8:**
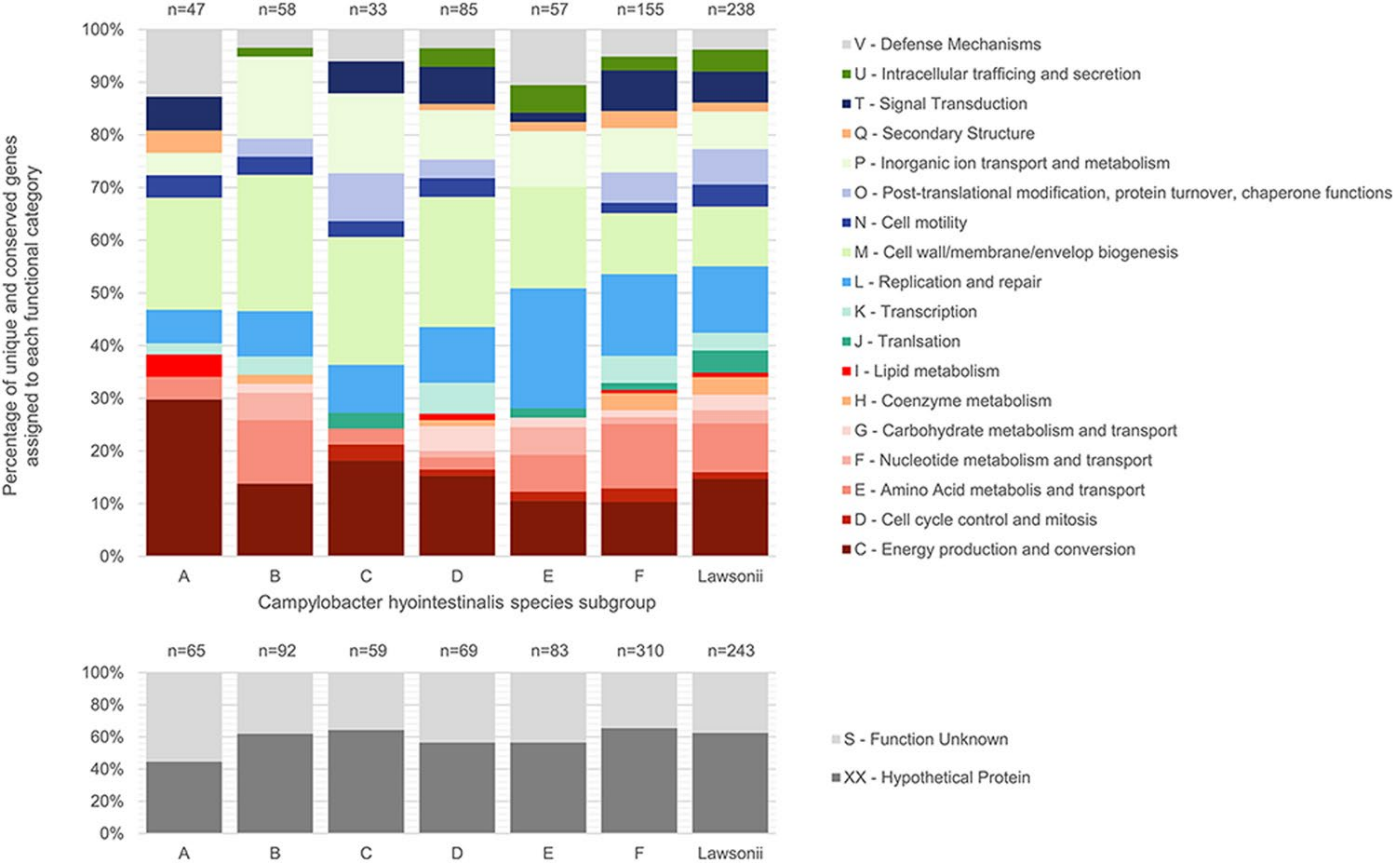
Accessory genome composition of the genomic clades of *C. hyointestinalis*. Clades are defined based on the congruence of the pangenome and phylogenetic analyses, as presented in Fig. 7. Gene groupings obtained by the use of get_homologues were used to define core and accessory genome components. Genes that are identified as unique to, and conserved within each defined clade are enumerated with respect to their functional category, as assigned using Eggnog-mapper^71^.

The atypical isolate S1499c provided the most extreme example of genomic plasticity in this dataset, with unique predicted coding genes that accounted for more than 10% of its total gene content. Examination of the contig-aligned distribution of the inherited gene content in this isolate showed that large genomic islands had been incorporated into its genome on at least one occasion and read-mapping and coverage analysis confirmed this was unlikely to be an artefact of genomic assembly (data not shown). Isolates were grouped into descriptive clade categories based on their predicted phylogenetic and pangenomic relationships (Fig. 7), and the functions of the characteristic genes from each clade (genes that were both conserved and unique) were derived (Fig. 8). For atypical isolate S1499c, the majority of these inherited genes had unknown function. Of those genes with predicted functions, a large proportion was associated with metabolic processes and, intriguingly, many genes had functions linked to the exchange of genetic material.

Characterisation of the predicted functions of clade-associated accessory genes showed that 25–40% of acquired genes had metabolic functions, and that gene acquisitions affecting cell membrane composition and signal transduction pathways occur frequently (Fig. 8). Additionally, multiple lineages had acquired genes that were associated with phage defence mechanisms such as restriction enzyme mediated, or CRISPR-mediated defence pathways.

To further explore the phage defence mechanisms employed by *C. hyointestinalis*, we characterised CRISPR elements and arrays from all available *C. hyointestinalis* genomes (Supplementary Table S4). All *C. hyointestinalis* subsp. *hyointestinalis* genomes contained components of at least one type I CRISPR-mediated defence system, whereas subspecies *lawsonii* had no CRISPR elements. Several lineages contained multiple copies of *cas1*, *cas2* and *cas9* genes. The number of CRISPR spacer sequences detectable in *C. hyointestinalis* genomes ranged from four to more than 100. Comparing the number of CRISPR arrays and the average spacer count per array to other *Campylobacter* genomes, we noted that the presence of CRISPR spacers is highly variable between *Campylobacter* species, and that *C. hyointestinalis* has both higher numbers of CRISPR array loci per genome and more individual spacer sequences per locus than most other species (Supplementary Figure 5).

Cytolethal distending toxin (CDT) is one of the main *Campylobacter*-associated protein toxins that has been linked to cytotoxicity^41^, and novel variants of CDT proteins have previously been reported in *C. hyointestinalis*^42^. We therefore compared gene similarity between all subunits of CDT identified within our genomic data to all publicly deposited gene sequences available in NCBI. We observed three distinct sub-categories for each of CDT subunits A, B and C (Supplementary Figure 6), and pseudogene variants caused by frame-shift mutations in subunits A and B (Supplementary Table 5).

## Discussion

In summary, we have shown that *C. hyointestinalis* subspecies *hyointestinalis* may be commonly isolated from livestock (sheep, cattle and deer) in New Zealand. Extended literature analysis shows that this is a common, global picture but that relatively little data exist to help us understand the epidemiology of this microbial species. Current data suggest that cattle, and possibly deer, are the main reservoir hosts of *C. hyointestinalis* subsp. *hyointestinalis* whereas *C. hyointestinalis* subsp. *lawsonii* is more commonly associated with pigs (also see ref.^36^). Thus, *C. hyointestinalis* infection in humans could be an occupational or environmental hazard in rural areas, or associated with contaminated food production. For example, two articles identified raw milk consumption as a likely source of infection^43,44^. Geographical context and regional farming practices are also likely to influence *C. hyointestinalis* distribution and prevalence. *C. hyointestinalis* infection has frequently been reported in association with human gastrointestinal illness (see Supplementary Data S1). Subspecies-level classification of human-infecting *C. hyointestinalis* is absent from all these published observations. Data from PubMLST suggests that human infection is most frequently attributed to subspecies *hyointestinalis*, however this data is mainly derived from higher income countries.

Of particular concern to human health, studies from South Korea^45,46^ have identified *C. hyointestinalis* in cases of non-*Helicobacter pylori* gastrointestinal adenocarcinoma. Virulence factors secreted by *H. pylori* during chronic infection of the gastric mucosa are a known risk factor for carcinogenesis^47^. In our data, all *C. hyointestinalis* genomes possessed the known *H. pylori*-associated virulence factor vacuolating cytotoxin A (*vacA*)^48^, but lacked other known virulence factors including both cytotoxin-associated gene A (*cagA*) and *oipA*. Further studies in areas of high human *C. hyointestinalis* infection incidence would be required to determine whether equivalent pathologies are associated with *C. hyointestinalis*. Cytolethal distending toxin is the most studied proteinaceous element known to be associated with cytotoxicity^41^. *C. hyointestinalis* isolates have previously been demonstrated to secrete multiple variants of CDT^49^. Our genomic analysis shows that many *C. hyointestinalis* variants carry multiple gene paralogues of CDT subunits A and C, and that gene truncation of specific subunit types is common, likely resulting in on/off CDT variant expression as a result of genomic frame shifts. We also observe that such virulence-related genes are commonly lost and acquired as part of the organism’s accessory pangenome, suggesting that these proteins’ roles in virulence are often mediated in response to changing environmental stimuli and selection pressures. Further studies of the mechanisms of virulence in C. hyointestinalis will be required to elucidate its role in both gastric and non-gastric disease.

Our analyses show an extremely high level of plasticity between *C. hyointestinalis* genomes. Single nucleotide polymorphism distances between isolates of the same sub-species commonly fell in the range of 10,000 to 20,000 SNPs, but only 10% of these were predicted to fall in genomic regions with no history of recombination. Mutation and recombination are both estimated to have been significant driving forces in the diversification of this species. Our analyses suggest that recombination/mutation rates are highly lineage specific, and that recombination may be more frequent between closely related lineages (see Fig. 7). The significant role of recombination in *C. hyointestinalis* evolution (and other *Campylobacter*, or indeed all other prokaryotic species) has direct implications for our ability to classify variants using simplistic phylogenetic techniques that rely on the validity of the tree of life hypothesis. Here, we noted that even within standard typing loci (MLST), recombination histories have significantly influenced ancestry to the extent that, when recombination is not accounted for, ancestral node support is dramatically diminished. Extending this to the scale of the whole genome, we estimate that less than 5% of genomic content can be considered to be part of the organism’s clonal frame, and thus that the tree of life hypothesis can be considered invalid for the majority of species-level or sub-species level comparative applications. As has been noted elsewhere^50^, this highlights the importance of techniques that accommodate recombination histories^39^, or the preference for network-based^51^ or ring-based^52^ analyses for comparative classifications of bacteria over tree-based methods.

Furthermore, although approximately 78% of the gene content of *C. hyointestinalis* can be considered as conserved (core genes), a large amount of the variation between subspecies variants can be attributed to components of the accessory genome. Although total genome size is relatively stable, even at subspecies level the *C. hyointestinalis* genome can be considered as open (Heap’s law coefficient = 0.21). Thus gene gain and loss are frequent in *C. hyointestinalis* evolution, but the temporally sparse nature of available data means that quantification of the timescale of these events is not currently possible. Studies of other microbial organisms have suggested that speciation may be promoted by large scale gene acquisition events followed by differential gene loss^53^, and our analyses suggest that this may also be the case within *C. hyointestinalis* lineages. Pangenome analysis demonstrated common variation in genes associated with metabolic processes, cell surface antigen presentation, DNA exchange processes and defence mechanisms. This is likely to reflect genomic adaptation to varying environmental selection pressures in dynamic niches. In such a situation, *C. hyointestinalis* pangenomes may undergo adaptive change^54^, acquiring genes as public goods^55^ from communities of organisms cohabiting each niche. Genes conferring a survival advantage will be subject to positive selection, and may even displace others via adaptation to the changing requirements for survival. In the case of genes like the CDT subunits (see previously), such dynamic selection will likely influence virulence characteristics, and thus disease associations will depend greatly on a strain’s natural history, even between members of the same species or subspecies. Quantification of gene exchange in such organisms is essential to correct interpretations for classification based on similarity between lineages: mosaic genomes that result from continuously sampled genes as public goods may result in misleading tree-like evolutionary patterns^55^.

Overall, due to its ubiquitous nature and low suspected virulence, *C. hyointestinalis* is likely to remain a background source of infection in both humans and animals. However, further epidemiological and molecular investigations are warranted, particularly in developing countries where less information is available. Like most ‘emerging *Campylobacter* spp.’, *C. hyointestinalis* is not uniformly associated with human disease. Whether this is due to organism-associated traits or diagnostic bias remains to be explored on a worldwide basis. Frequent homologous and non-homologous recombination adds considerable complexity to the challenges of taxonomic classification and source attribution. This limits what can be generalised about the important traits of *C. hyointestinalis* such as its virulence, host-preference and metabolic capacity. Natural histories will play an important role in determining the genomic composition of each variant of *C. hyointestinalis* and thus studies of local variants are required to address local issues in both epidemiology and disease.

## Materials and Methods

### Literature survey

An initial list of publications for systematic review was compiled from the search results of both PubMed and Google Scholar, searching for any article in which the phrase “*Campylobacter hyointestinalis*” appeared at any position in the text. This search returned more than 1500 published research articles and reports. Further search inquiries were performed for any apparently relevant publications that were cited by articles that appeared in the original list. Articles were manually curated and assessed for their relevance. Only articles that reported original observations of *C. hyointestinalis*, and employed robust strategies to identify this particular species, were included in the final literature review (summarised in Supplementary Data 1).

### Microbiology

Samples were collected from subprojects in our ongoing*Campylobacter* surveillance programme. In total, six hundred and sixty ruminant faecal samples were collected on 33 farms across the Manawatu district between 2008 and 2016, after pooling this represented a total of 225 independent samples (see Supplementary Table S1). Of these, 16 were dairy farms, seven were sheep farms, nine were sheep and beef farms and one was a red deer (*Cervus elaphus*) farm. Isolations from deer were performed in order to examine potential human disease sources, and therefore culture conditions were selected to mirror those used in human *Campylobacter* isolation. Sheep and cattle samples were collected as part of a wider, ongoing project which uses alternative culture conditions to assess the bacterial diversity of infections in ruminant livestock. Swabs were taken from fresh faeces on the ground. For deer, swab tips were inoculated into 3 ml Bolton Broth (Lab M, Bury, United Kingdom) and incubated in a microaerobic (85% nitrogen, 5% oxygen, 10% carbon dioxide) incubator (Don Whitley, Shipley, England) for 48 hours at 42 °C. Modified Cefoperazone Charcoal Deoxycholate Agar (mCCDA) plates (Fort Richard, Auckland, New Zealand) were inoculated with the broth and incubated microaerobically for 48 hours at 42 °C. For cattle and sheep, duplicate pools of four swabs were inoculated into i) 20 ml Bolton Broth and incubated as for the deer swabs and ii) 20 ml CAT Broth (Oxoid, Hampshire, England) and incubated in a hydrogen-enriched microaerobic (82% nitrogen, 3% oxygen, 10% carbon dioxide, 5% hydrogen) incubator (Don Whitley, Shipley, England) for 48 hours at 37 °C. mCCDA plates were inoculated with Bolton broth and incubated microaerobically for 48 hours at 42 °C. CAT plates (Fort Richard, Auckland, New Zealand) were inoculated with CAT broth and incubated in a hydrogen-enriched microaerobic incubator for 48 hours at 37 °C. Where growth was observed, two discrete colonies were removed and grown on separate Colombia horse blood agar plates (Fort Richard, Auckland, New Zealand) for 48 hours in the same atmosphere and temperature as the original plate.

### Molecular Biology

Crude DNA extracts from colonies resembling *Campylobacter* were made and *Campylobacter* genus PCR performed using the primers of Linton^56^ as described by Bojanić *et al*.^57^. Positive isolates were speciated by PCR using the primers of Stucki^58^ for *C. jejuni*, those of Denis^59^ for *C. coli* and those of Linton^56^ for *C. fetus*, *C. hyointestinalis*, *C. lari* and *C. upsaliensis*, as described in Bojanić *et al*.^57^. Purified genomic DNA was extracted using a QIAamp Mini kit (Qiagen, Hilden, Germany) following the manufacturer’s instructions with minor modifications. Genomic DNA was prepared for sequencing using the Nextera XT library kit (Illumina, San Diego, USA). Libraries were sequenced on an Illumina HiSeq using 2 × 250 bp paired end sequencing by the Massey Genome Service (Palmerston North, New Zealand), part of New Zealand Genomics Ltd.

### Bioinformatics, population genetics and evolutionary analysis

Illumina reads were trimmed using a combination of in-house software and Trimmomatic^60^, assembled using SPAdes version 3.9.0^61^ and annotated using Prokka version 1.11^62^ as part of the Nullarbor pipeline (https://github.com/tseemann/nullarbor), which was also used to extract assembly statistics which are presented in Supplementary Table S2. Assembled draft genomes were deposited to NCBI with accession numbers NIQE00000000 – NIQT00000000.

Genus-level whole genome MLST was performed using the Genome Profiler software package^63^ under default settings and the complete genome of *C. hyointestinalis* LM9260^36^ as a reference. Network diagrams were computed from the core genome alignment output of Genome Profiler using Splitstree version 4.14.4^64^.

Seven-gene MLST alignments and isolate data were obtained for all isolates recorded in PubMLST^65^. Minimum spanning analysis was generated from MLST alignments using the PopArt software package (http://popart.otago.ac.nz), pairwise p-distances were calculated using a Jukes-Cantor substitution model in the MATLAB R2016b Statistics and the Machine Learning Toolbox. New seven-gene allele sequences were verified by PCR amplification^66^ and Sanger sequencing (Supplementary Data S3).

Sites of recombination and recombination to mutation ratios were estimated using Gubbins^39^. Phylogenetic analysis of recombination stripped alignments was performed in BEAST2^67^ with an MCMC chain length of 100,000,000 using an HKY substitution model accounting for invariant sites, a strict molecular clock and a Yule population size model. Convergence of the phylogenetic Bayesian inference was verified in Tracer v1.6 (http://beast.bio.ed.ac.uk/Tracer), with all estimated parameters returning estimated sample sizes values greater than 200 after a 10% burn-in.

Whole genome comparison and the assignment of clusters of orthologous genes for genomes belonging to the *C. hyointestinalis* species was performed using get_homologues^68^ and the OrthoMCL clustering algorithm^69^. Prior to get_homologues execution, all reference genome datasets acquired from public data repositories were reannotated using Prokka version 1.11^62^ in order to ensure similar annotation schemes between comparisons. Protein functional groups were assigned to accessory genes using HMMER3 (http://hmmer.janelia.org/) to query BactNOG, the bacterial subset of the EggNOG4.5 database^70^ through the online EggNOG-mapper tool^71^. Pangenome composition was explored and visualised using the hierarchicalSets package in R^72^. Tanglegrams were generated using the dendextend package for R^73^. Gene gain and loss events were estimated using the Count software package^74^ based on the birth and death model described by Csűrös and Miklós^53^, and allowing variation in both the gain:loss and duplication:loss ratios.

For gene-based analysis of the cytolethal distending toxin (CDT) subunits; relevant clusters of orthologous genes were identified after functional characterisation in EggNog-mapper. Reference protein sequences were obtained from NCBI using the search terms “cytolethal distending toxin” and “organism contains ‘Campylobacter’”. Sequences were manually curated, aligned using ClustalW, and UPGMA tree representations of gene similarity were generated in the Geneious software package (Biomatters v.10.0.8). CRISPR spacer regions were detected using the CRISPRfinder^75^ software package, and repeats were enumerated per speices using custom-written scripts in MATLAB R2016b.

### Accession numbers

Assembled draft genomes were deposited to NCBI with accession numbers NIQE00000000–NIQT00000000.

## Electronic supplementary material

Supplementary Information

Supplementary data S2
