# Supplementary material for: Updating the genomic taxonomy and epidemiology of Campylobacter hyointestinalis
Source: Sci Rep. 2018 Feb 5;8:2393. doi: 10.1038/s41598-018-20889-x (PMC5799301; doi:10.1038/s41598-018-20889-x)
Supplement: Supplementary file 1 — Supplementary Information [file 41598_2018_20889_MOESM1_ESM.pdf]

Supplementary Information For:

Updating the genomic taxonomy and epidemiology of *Campylobacter hyointestinalis*.

David A Wilkinson[1*], Andrew J O'Donnell[2], Rukhshana N Akhter[1], Ahmed Fayaz[1], Hamish J Mack[1], Lynn E Rogers[1], Patrick J Biggs[1,3], Nigel P French[1,3], Anne C Midwinter[1]

1 Molecular Epidemiology and Public Health Laboratory (*m*EpiLab), Institute of Veterinary, Animal and Biomedical Sciences, Massey University, Palmerston North, New Zealand

2 Faculty of Veterinary Science, University of Liverpool, Leahurst, Cheshire, CH64 7TE. United Kingdom

3 New Zealand Food Safety Science and Research Centre, Massey University, Palmerston North, New Zealand

* Denotes corresponding author.

Correspondence: E-Mail: D.A.Wilkinson@massey.ac.nz

Telephone: +64 (06) 356 9099 extn 85752

Correspondence for proofs: Molecular Epidemiology and Public Health Laboratory (*m*EpiLab), Massey University, Tennent Drive, Private Bag 11-222, Palmerston North, 4442.

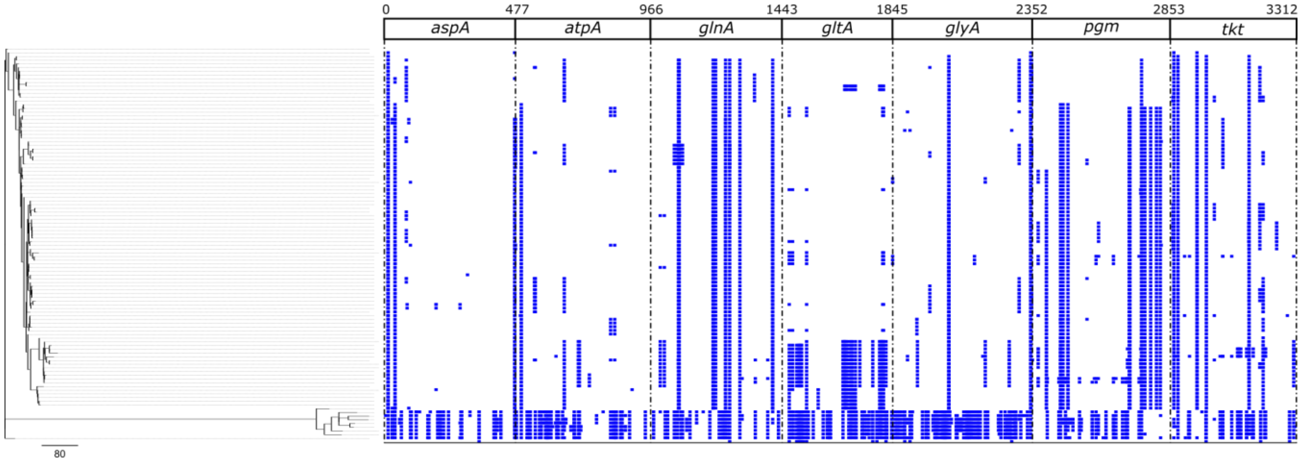

Supplementary figure 1: Prediction of recombinant sites within the *C. hyointestinalis* seven-gene MLST scheme

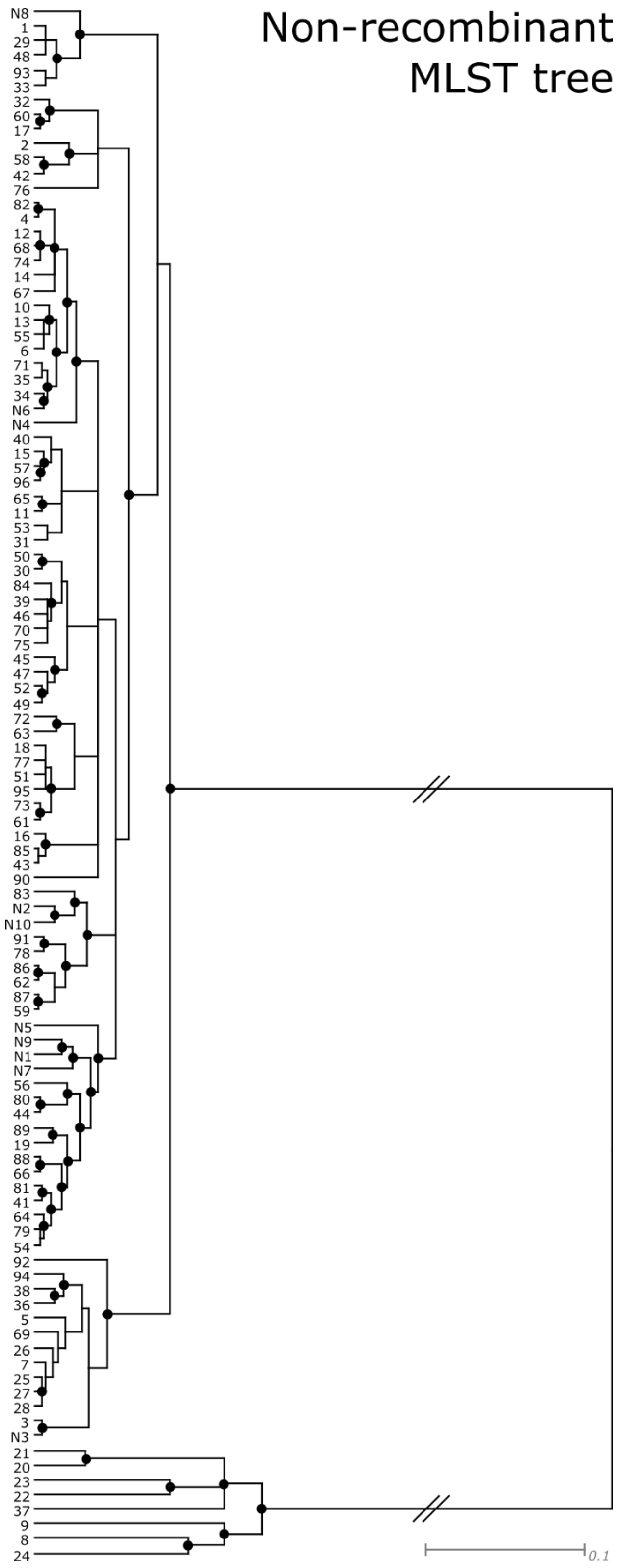

Supplementary Figure 2: Recombination-stripped MLST gene tree for all reported C. hyointestinalis sequence types in PubMLST. New allelic profiles were assigned as in Supplementary Table S3. Circular points indicate nodes with posterior probabilities >0.95.

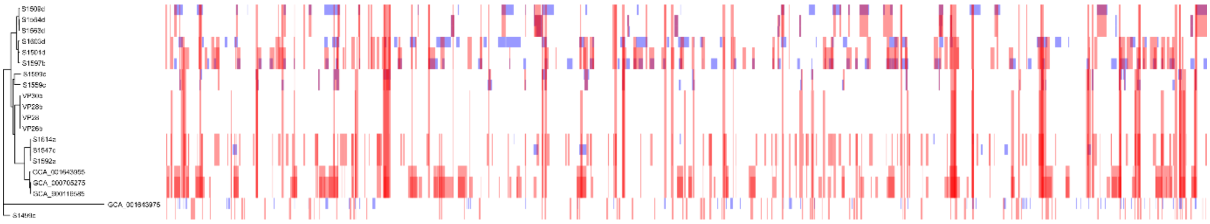

Supplementary Figure 3: Prediction of recombinant sites within the *C. hyointestinalis* whole genome gene alignment. The presented phylogeny shows the predicted relationship between isolates based on non-recombinant regions. Blue bars highlight genomic loci that are predicted to have undergone recombination within single genomes, whereas red bars denote recombinant regions that are predicted to be shared between multiple genomes.

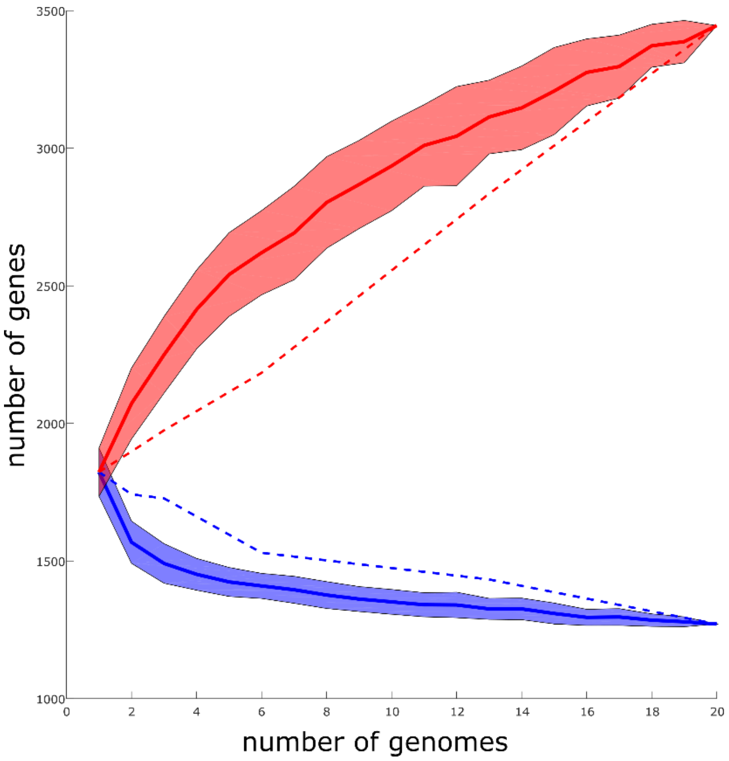

Supplementary Figure 4: Rarefaction analysis of the core genome (blue) and pangenome (red) of *C. hyointestinalis* genomes. Dotted lines indicate the rate of core/pangenome decay when constrained to genome clusters defined by the hierarchical clusters method depicted in main figure 5.

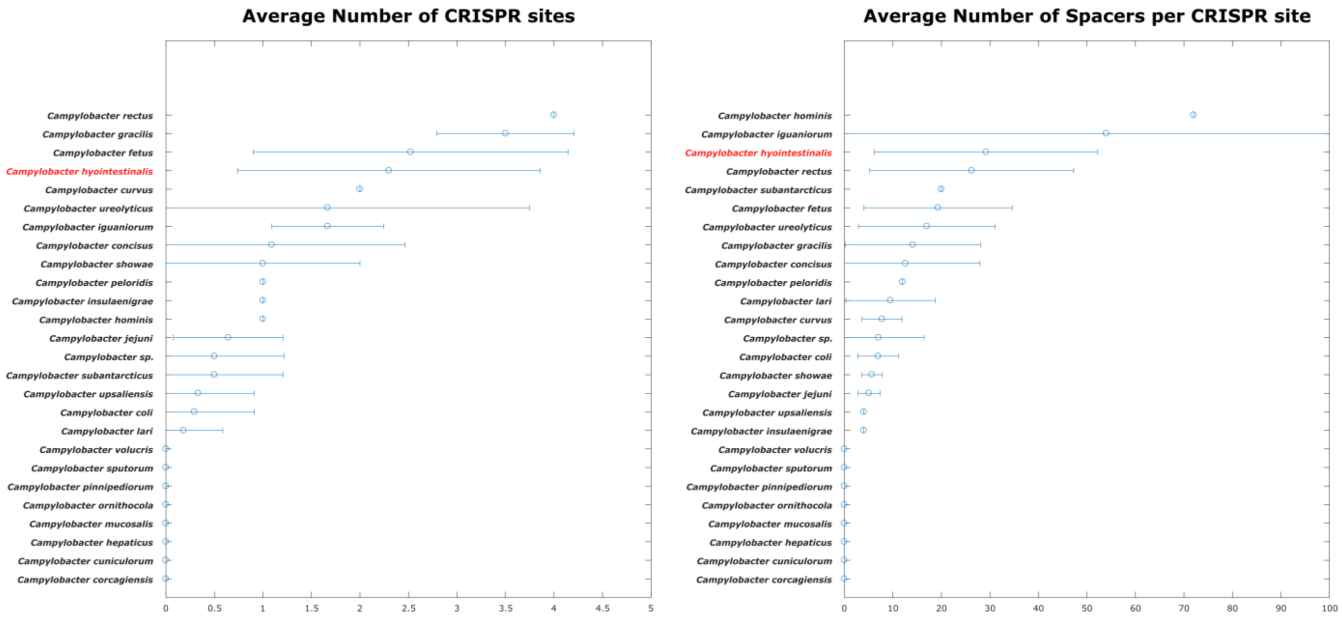

Supplementary Figure 5: Average +/- standard deviation of the number of CRISPR sites (left) and number of spacer regions (right) per *Campylobacter* species, as identified from 1,884 available genomes from across the *Campylobacteraceae* family.

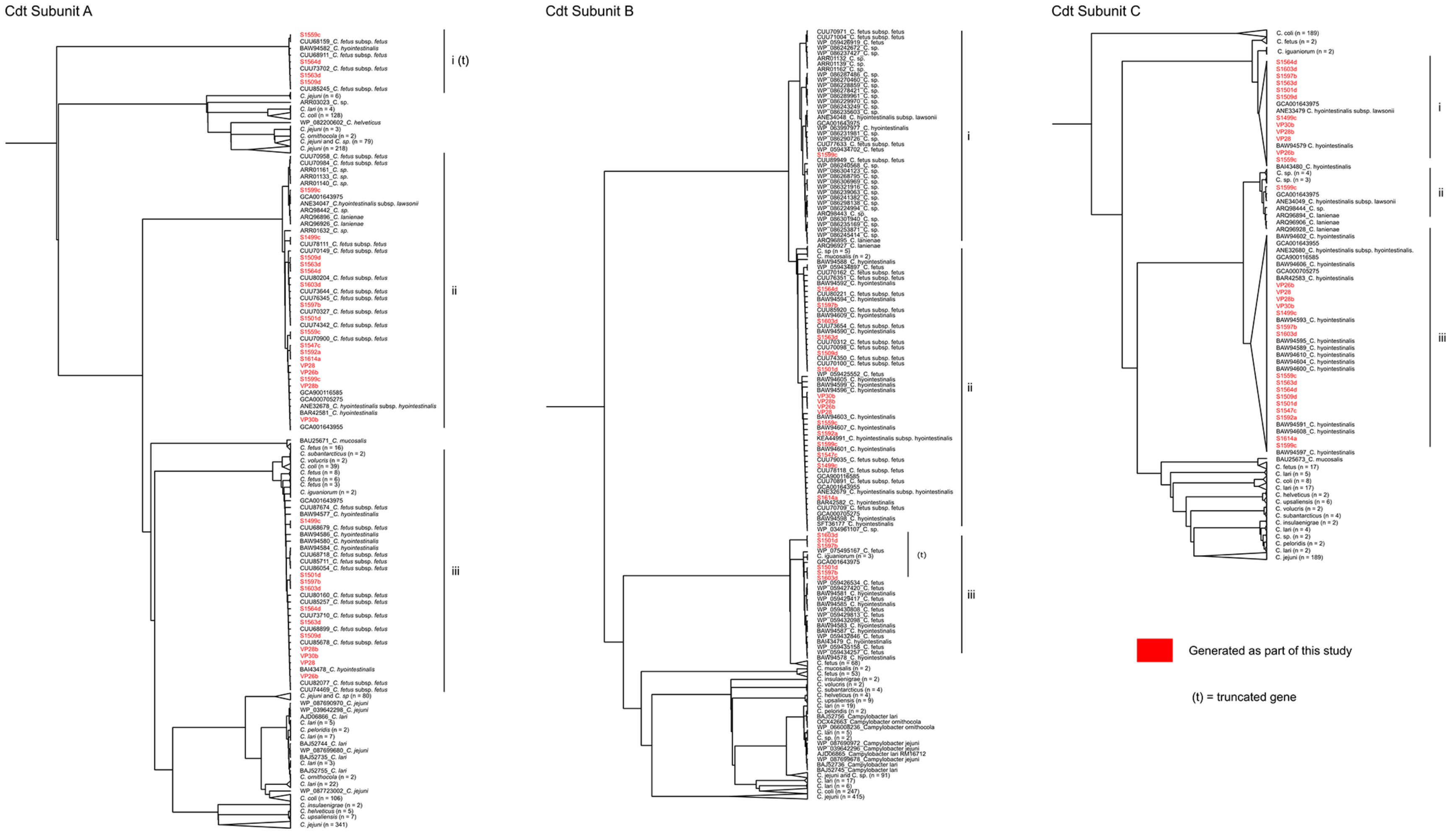

Supplementary Figure 6: UPGMA gene trees of cytolethal distending toxin (CDT) subunits identified within genomes of *C. hyointestinalis* generated as part of this study (red), and compared to all available CDT data available in the NCBI protein repository (black). Groups are defined based on phylogenetic relatedness, as shown, for each subunit.

Species	Pooled swabs	# tested (# swabs)	# samples from which *Campylobacter* was isolated (%)	# identified with *C. jejuni/ C. coli* (%)	# identified with *C. hyointestinalis* (%)
Cattle	Y	77 (308)	73 (94%)	57 (74%)	28 (36%)
Sheep	Y	68 (272)	35 (51%)	29 (42%)	6 (9%)
Deer	N	80	9 (11%)	2 (3%)	8 (18%)
TOTAL	-	225	117 (52%)	88 (39%)	48 (21%)

Supplementary Table S1: Diagnostic results for *C. jejuni*/*C. coli* and *C. hyointestinalis* from cattle, sheep and deer faeces.

Isolate	Accession Number	Contigs	Average Fold-Coverage	Genome Size (Mbp)	GC Content (%)	N50	Coding regions	rRNA copies	tRNA copies
S1499c	NIQE00000000	52	73	1.93	35.0	178581	2049	2	42
S1501d	NIQF00000000	39	71	1.73	35.4	217497	1809	2	42
S1509d	NIQG00000000	37	83	1.78	35.0	197085	1857	2	42
S1547c	NIQH00000000	79	94	1.81	35.9	145507	1891	2	42
S1559c	NIQI00000000	29	133	1.78	36.2	556818	1864	2	43
S1563d	NIQJ00000000	51	85	1.76	34.8	78373	1865	2	42
S1564d	NIQK00000000	76	71	1.78	34.4	83731	1868	2	40
S1592a	NIQL00000000	42	83	1.81	36.1	175279	1901	2	42
S1597b	NIQM00000000	57	98	1.77	34.7	308874	1859	2	42
S1599c	NIQN00000000	73	57	1.78	36.3	439663	1841	2	42
S1603d	NIQO00000000	41	89	1.72	34.5	255033	1799	2	42
S1614a	NIQP00000000	50	112	1.81	36.1	195895	1906	2	42
VP26b	NIQQ00000000	27	58	1.70	37.0	242619	1713	2	42
VP28	NIQR00000000	44	98	1.70	36.9	194759	1718	2	42
VP28b	NIQS00000000	37	55	1.70	36.6	309452	1719	2	42
VP30b	NIQT00000000	63	48	1.70	37.0	118626	1720	2	42

Supplementary Table S2: Genome assembly statistics

	aspA	*atpA*	*glnA*	*gltA*	*glyA*	*pgm*	*tkt*	ST
S1603d	1	1	NEW_1	4	10	NEW_1	10	N1
S1564d	1	1	NEW_2	4	8	4	12	N2
S1547c	1	2	3	3	3	3	11	N3
S1592a	1	2	3	3	3	3	11	N3
S1614a	1	2	3	3	3	3	11	N3
S1599c	1	NEW_3	2	4	1	18	NEW_1	N4
S1597b	18	NEW_2	1	4	10	NEW_2	6	N5
S1559c	19	1	5	4	1	18	6	N6
S1499c	NEW_1	NEW_1	NEW_3	21	9	NEW_1	26	N7
VP26b	NEW_2	NEW_4	NEW_4	19	1	1	1	N8
VP28	NEW_2	NEW_4	NEW_4	19	1	1	1	N8
VP28b	NEW_2	NEW_4	NEW_4	19	1	1	1	N8
VP30b	NEW_2	NEW_4	NEW_4	19	1	1	1	N8
S1501d	5	1	NEW_1	4	8	NEW_1	6	N9
S1509d	6	1	NEW_1	4	8	4	12	N10
S1563d	6	1	NEW_1	4	8	4	12	N10

Supplementary Table S3: MLST-scheme allele definitions and ST-profile definitions for sequenced isolates from this study. Pre-existing allele definitions were taken from the PubMLST website. The described isolate is available within PubMLST.

Strain ID	Host	Lineage (Fig. 7)	Subsp.	Cas1	Cas2	Cas3	Cas4	Cas5	Cas6	Cas7	Cas8	Cas9	CRISPRs	Spacers
VP26b	Deer	A	Hyo.	1	1	1	-	1	1	-	-	-	2	30, 36
VP28	Deer	A	Hyo.	1	1	1	-	1	1	-	-	-	3	36, 25, 7
VP28b	Deer	A	Hyo.	1	1	1	-	1	1	-	-	-	3	36, 25, 7
VP30	Deer	A	Hyo.	1	1	1	-	1	1	-	-	-	3	36, 25, 7
S1559c	Cow	B	Hyo.	2	2	1	-	1	1	-	-	2	2	46, 56
S1599c	Cow	B	Hyo.	1	1	1	-	1	1	-	-	-	3	28, 18, 2
S1509d	Cow	D	Hyo.	1	1	1	-	1	1	-	-	-	2	27, 37
S1563d	Cow	D	Hyo.	1	1	1	-	1	1	-	-	-	1	45
S1564d	Cow	D	Hyo.	1	1	1	-	1	1	-	-	-	3	49, 7, 13
S1501d	Cow	C	Hyo.	1	1	1	-	1	1	-	-	-	2	32, 46
S1597b	Sheep	C	Hyo.	1	1	1	-	1	1	-	-	-	4	33, 62, 19, 3
S1603d	Cow	C	Hyo.	1	1	1	-	1	1	-	-	-	1	51
GCF_000705275	Pig	E	Hyo.	1	1	1	-	1	1	-	-	-	4	3, 90, 28, 61
GCF_001643955	Human	E	Hyo.	2	2	1	1	1	1	1	-	1	2	6, 113
GCF_900116585	Pig	E	Hyo.	1	1	1	-	1	1	-	-	-	7	3, 2, 19, 6, 16, 17, 24
S1499c	Cow	F	Hyo.	1	1	1	-	1	-	-	-	1	1	4
S1547c	Sheep	E	Hyo.	3	2	1	-	1	1	-	-	3	1	18
S1592a	Cow	E	Hyo.	3	2	1	-	1	1	-	-	3	1	23
S1614a	Cow	E	Hyo.	3	2	1	-	1	1	-	-	3	1	23
GCF_001643975	Pig	E	Law.	-	-	-	-	-	-	-	-	-	0	-

Supplementary Table S4: Summary of the number of CRISPR/Cas components, spacers and spacer lengths identified within each genome of *C. hyointestinalis*.

		CDT Subunit A			CDT Subunit B			CDT Subunit C		
Strain ID	Subsp.	i	ii	iii	i	ii	iii	i	ii	iii
VP26b	Hyo.		1	1		1		1		1
VP28	Hyo.		1	1		1		1		1
VP28b	Hyo.		1	1		1		1		1
VP30b	Hyo.		1	1		1		1		1
S1559c	Hyo.	1 (t)	1			1		1		1
S1599c	Hyo.		2		1	1			1	1
S1509d	Hyo.	1 (t)	1	1		1		1		1
S1563d	Hyo.	1 (t)	1	1		1		1		1
S1564d	Hyo.	1 (t)	1	1		1		1		1
S1501d	Hyo.		1	1		1	1 (t)	1		1
S1597b	Hyo.		1	1		1	1 (t)	1		1
S1603d	Hyo.		1	1		1	1 (t)	1		1
S1499c	Hyo.		1	1		1		1		1
S1547c	Hyo.		1			1				1
S1592a	Hyo.		1			1				1
S1614a	Hyo.		1			1				1
GCF_000705275	Hyo.		1			1				1
GCF_001643955	Hyo.		1			1				1
GCF_900116585	Hyo.		1			1				1
GCF_001643975	Law.		1	1	1		1	1	1	

Supplementary Table S5: Summary of Cytolethal distending toxin (CDT) subunit types identified within *C. hyointestinalis* genomes sequenced as part of this project. (t) indicates that the gene has been truncated. Subunit types are defined as displayed in Supplementary figure 6.

Supplementary Data For:

Updating the genomic taxonomy and epidemiology of *Campylobacter hyointestinalis*.

David A Wilkinson[1*], Andrew J O'Donnell[2], Rukhshana N Akhter[1], Ahmed Fayaz[1], Hamish J Mack[1], Lynn E Rogers[1], Patrick J Biggs[1,3], Nigel P French[1,3], Anne C Midwinter[1]

1 Molecular Epidemiology and Public Health Laboratory ([m]EpiLab), Institute of Veterinary, Animal and Biomedical Sciences, Massey University, Palmerston North, New Zealand

2 Faculty of Veterinary Science, University of Liverpool, Leahurst, Cheshire, CH64 7TE. United Kingdom

3 New Zealand Food Safety Science and Research Centre, Massey University, Palmerston North, New Zealand

* Denotes corresponding author.

Correspondence: E-Mail: D.A.Wilkinson@massey.ac.nz

Telephone: +64 (06) 356 9099 extn 85752

Correspondence for proofs: Molecular Epidemiology and Public Health Laboratory ([m]EpiLab), Massey University, Tennent Drive, Private Bag 11-222, Palmerston North, 4442.

Supplementary Data 1: Table of references from the systematic review of *Campylobacter hyointestinalis* publications

Ref	Pub Year	Country	Isolation Method	Inc. Temp.	Typing Method	Source	# Tested	# Hyo	Disease	Comments
THIS STUDY	2017	New Zealand	Bolton broth; mCCDA	42	Linton PCR; WGS	Deer faeces	80	14	-	-
1	2015	South Korea	Solid selective culture medium	42	MALDI-TOF (Bruker); 16S PCR sequencing	Human stool	1	1	Diarrhoea	Elderly patient, Immunocompromised
2	2015	Italy	CAT agar<br>Preston broth; Karmali agar	37	Multiplex PCR; Linton PCR sequencing	Dog faeces	171	3	-	Isolated infection (same shelter)
						Cat faeces	102	0	-	-
3	2015	Thailand	mCCDA	37	16S PCR sequencing; HipO gene detection	Human stool	2500	1	Diarrhoea	-
4	2014	Canada	-	-	qPCR	Cattle offal or meat	309	24	-	Weak correlation between *C.hyointestinalis* presence and total bacterial load.
5	2014	Canada	Bolton broth; Karmali agar	42	Multiplex PCR	Cattle faeces	935	171	-	Dairy cows
6	2014	Peru	Cary-Blair transport medium; Blood-free Campylobacter selective medium	42	Duplex PCR; 16S PCR sequencing	Human stool	150	5	-	-
		Tanzania	Cary-Blair transport medium; Solid selective culture medium	42	Duplex PCR; 16S PCR sequencing	Human stool	138	17	-	Pigs are commonly raised in houses at this location
		Bangladesh	Cary-Blair transport medium; Campy-BAP	42	Duplex PCR; 16S PCR sequencing	Human stool	144	6	-	-
7	2013	Japan	mCCDA<br>Preston Broth; mCCDA	42	API Campy; 16S PCR sequencing	Boar faeces	121	21	-	Nalidixic acid resistant
			mCCDA<br>Preston Broth; mCCDA	42	API Campy; 16S PCR sequencing	Deer faeces	128	0	-	-
8	2013	Italy	Bolton Broth; mCCDA; Preston selective medium; Skirrow selective medium	37	Multiplax PCR; Duplex PCR; 16S PCR sequencing	Raw Buffalo milk	14	0	-	Milk in-line filter was tested.
			Bolton Broth; mCCDA; Preston selective medium; Skirrow selective medium	37	Multiplax PCR; Duplex PCR; 16S PCR sequencing	Raw cow milk	182	8	-	Milk in-line filter was tested.
9	2013	The Netherla	NS	NS	MLST	Dog faeces	315	25	-	*C. hyointesinalis* classification also includes *C.*

		nds								*fetus*
			NS	NS	MLST	Cat faeces	109	0	-	C. hyointesinalis classification also includes *C. fetus*
10	2012	Ireland	mCCDA	42	EntericBio system; Species-specific Uniplex PCR; 16S PCR sequencing	Human stool	436	4	Diarrhoea	-
11	2011	South Korea	-	-	16S PCR sequencing	Human gastro-intestinal tract	20	12	Unspecified Gastric disease	All Giemsa-positive histological samples from endoscopy.
12	2011	Spain	Preston broth; Campylosel plates	42	Multiplex PCR; 16S PCR sequencing	Cattle faeces	206	86	-	-
						Sheep faeces	120	2	-	-
						Pig faeces	17	2	-	-
13	2011	Canada	-	-	Species-specific Uniplex PCR; 16S PCR sequencing	Human stool	442	8	Diarrhoea	*C. hyointestinalis* was not detected by culture, by detected in low prevalence by PCR.
						Human stool	58	0	-	-
14	2011	Ireland	-	-	EntericBio system; Species-specific Uniplex PCR; 16S PCR sequencing	Human stool	7194	5	Diarrhoea	-
15	2010	South Korea	-	-	16S PCR sequencing	Human gastro-intestinal tract	51	1	Gastric adenocarcinomas	-
16	2009	Nigeria	mCCDA	42	Phenotypic characterisation and Biotyping	Cattle faeces	126	4	-	-
17	2007	Finland	mCCDA<br>Bolton broth; mCCDA	41.5	Serotyping; PFGE	Cattle faeces	952	103	-	*C. hyointestinalis* only detected after enrichment Monthly prevalence ranged from 5 % - 23 %
						Cattle offal or meat	948	2	-	*C. hyointestinalis* only detected after enrichment
18	2006	Austria	-		Multiplex PCR	Chicken faeces	31	0	-	-
						Human faeces	10	0	-	-
						Pig faeces	2	1	-	-
19	2006	Canada	mCCDA; cCCDA; eCCDA	42	Phenotypic characterisation; Uniplex PCR	Cattle faeces	7738	4	-	Cattle were systematically treated with antibiotics for

										production. Ciprofloxacin and erythromycin resistant *C. hyointestinalis* variants were isolated.
20	2005	Canada	brain heart infusion broth; mCCDA; cCCDA; eCCDA	42	23S PCR sequencing	Cattle faeces	3283	1518	-	Cattle were experimentally treated with antibiotics. Tetracycline, erythromycin and ampicillin resistant *C. hyointesinalis* variants were isolated
21	2004	Canada	-	-	16S PCR sequencing	Cattle faeces	299	23	-	60 animals, maintained in experimental feed lot, sampled on 5 occasions
22	2003	Canada	-	-	Uniplex PCR	Cattle faeces	382	30	-	-
23	2004	UK	Campylobacter enrichment broth; Campylobacter blood-free agar (CA supplement)	42	Uniplex PCR	Cattle faeces	1014	7	-	-
						Bird faeces	180	1		
						Sheep faeces	24	0		
						Other wildlife faeces	271	0		
						Environmental sample	1152	0		
24	2002	Finland	CAT broth; CAT agar	37	Phenotypic characterisation; SDS-PAGE; DNA-DNA hybridisation; 23S RFPCR, PFGE	Reindeer faeces	399	24	-	All isolates Nalidixic acid resistant
25	2002	Austria	mCCDa	37; 42	Phenotypic characterisation; API Campy; 16S PCR sequencing; PFGE	Human stool	1	1	Chronic Diarrhoea	Elderly patient. Infected from a pig. All isolates Nalidixic acid resistant
						Pig faeces	16	2	-	All isolates Nalidixic acid resistant
26	1985	USA	NS	37	Phenotypic characterisation; Serotyping	Pig illeum	30	10	Proliferative enteritis	-
27	1986	USA	Brucella agar + sheep blood	35	Phenotypic characterisation; DNA-DNA hybridisation	Human stool	1	1	Diarrhoea	-
28	1986	USA	Mueller-Hinton agar, 5 % defibirinated sheep blood	37	Phenotypic tests; Microagglutination Test	Pig illeum	14	5	Chronic diarrhoea	-
29	1987	USA	NS	NS	Phenotypic tests; Fatty-acid content chromatography; DNA-	Human stool	4	4	Chronic diarrhoea	All isolates Nalidixic acid resistant

					DNA hybridisation					
30	1987	Australia	CSB; CA; aerobic incubation	NS	Phenotypic tests	Deer faeces	24	23	Chronic diarrhoea	All isolates Nalidixic acid resistant
31	1988	France	Rogol broth enrichment medium; Columbia blood agar + horse defibrinated blood + Skirrow	42	Phenotypic tests	Human stool	1	1	Diarrhoea	Immunocompromised patient
32	1988	UK	-	-	Phenotypic tests; Seroloical analysis	Cattle faeces	497	165	-	Study classified isolates, did not measure prevalence of infection
						Sheep faeces	51	0	-	Study classified isolates, did not measure prevalence of infection
33	1989	Turkey	Skirrow selective agar	37	Phenotypic tests	Cattle faeces	14	12	Diarrhoea	-
34	1992	USA	-	-	SDS-PAGE, RFPCR, DNA-DNA hybridisation	Non-human primate faeces	18	7	-	Study classified isolates, did not measure prevalence of infection
35	1993	Japan	Preston enrichment broth; Skirrow agar	37	Phenotypic tests	Cattle faeces	94	22	-	*C. hyointestinalis* was only isolated in enriched broth, due to low bacterial loads. Calves showed higher levels of *C. jejuni* infection. Adult cows had equivalent *C. jejuni*, *C. fetus* and *C. hyointestinalis* prevalence.
36	1997	The Netherlands	CCD broth, CCDA	42	Phenotypic tests, SDS PAGE, RAPD	Shellfish (marine water)	59	1	-	-
37	1998	UK	CAT broth; CAT agar, mCCDA, Karmali agar	37	Phenotypic tests, API Campy	Cattle faeces	158	49	-	-
38	1998	UK	mCCDA	37	Phenotypic tests; 16S PCR sequencing	Human stool	200	1	Gastroenteritis	-
39	1999	Switzerland	Campylosel agar	37	DNA-DNA hybridisation	Cattle faeces	1521	203	-	Animals were all calves.
40	1999	UK	mCCDA	41-42	Uniplex PCR, PCR-ELISA	Human stool	3738	3	Gastroenteritis	-
41	2000	Japan	mBlaser	42	Phenotypic tests, SDS PAGE, RAPD	Elephant faeces	1	1	-	All animals were zoo animals
			CEM, mBlaser; mBlaser	37, 42	Phenotypic tests, SDS PAGE, RAPD	Non-Human primate faeces	15	10	-	All animals were zoo animals

42	2009	Finland	Bolton broth; mCCDA	41.5	Phenotypic tests, PFGE	Cattle faeces	340	52	-	-
43	2002	Italy	Preston broth + horse blood + preston supplement, mCCDA	42	Phenotypic tests	Cattle faeces and meat	890	16		Numbers are approximate, isolation was carried out on pooled samples, and typing was not completed for all samples
						Pig faeces and meat	1040	16		Numbers are approximate, isolation was carried out on pooled samples, and typing was not completed for all samples
44	2006	Belgium	Butzler agar + sheep blood	42	Phenotypic tests	Human stool	73,136	2	Gastroenteritis	-
45	2002	Denmark	Preston broth; mCCDA	41.5	Phenotypic tests, Serotyping, PFGE	Cattle faeces	332	>1	-	Exact numbers not specified for *C. hyointestinalis*
46	2014	UK	Campylobacter enrichment broth + CVTC; CSA agar	37	16S PCR sequencing, multiplex PCR, uniplex PCR, duplex PCR	Cattle faeces	3300	380	-	-
47	2012	Pan-Europe	NS	NS	NS	Mixed animal samples	1624	15	-	Study focuses on antimicrobial resistance in isolates, not on typing and host-association.
48	2012	Nigeria	Skirrow broth; Skirrow agar	37	Phenotypic tests	Cattle cervico-vaginal mucus	36	3	-	-
49	2011	Australia	-	-	16S PCR Sequencing	Human gastrointestinal tract	132	4	Inflammatory Bowel Disease; Crohn's Disease; Healthy	-
50	2006	Finland and Norway	Preston broth, Preston agar	37	Phenotypic tests, ApiCampy	Reindeer faeces	2243	1	-	-
51	2012	USA	Filtration, ABA agar	37	MALDI-TOF, Multiplex PCR, 16S PCR sequencing, MLST	Pig faeces	30	1	-	-
52	2008	USA	-	-	16S metagenomics	Non-human primate faeces	1	1	-	Species classification is broad
53	2014	The Netherlands	Sheep blood agar, CCD agar, Preston agar, Skirrow agar, membrane filtration	37	Uniplex PCR, 16S PCR sequencing, AFLP	Tortoise faeces	154	9	-	-
54	2011	Hungary	mCCDA	NS	16S PCR Sequencing	Pig faeces	480	3	-	-

55	1999	UK	Ambig.	Ambig.	Phenotypic tests, PFGE	Cattle faeces	26	4	-	-
56	2010	Rwanda	Campylobacter thioglycollate enrichment medium, Campylobacter-selective medium	2-4, 42	RAPID PCR Sequencing	Human stool	18	1	-	-
57	2012	Tanzania	Ambig.	NS	16S PCR Sequencing	Human stool	205	1	NS	-
58	2005	Portugal	-	-	16S PCR Sequencing	Human stool	807	1	-	Study classified existing clinical isolates
59	2003	Belgium	Preston broth, mCCDA	42	16S PCR Sequencing	Cattle faeces	2	1	-	-
60	2015	Thailand	-	-	16S PCR Sequencing, PFGE, DNA-DNA hybridisation	Human stool	2500	1	Diarrhoea	-
61	2015	Nigeria	mCCDA	42	Phenotypic tests	Human stool	94	5	-	-
						Pig faeces	278	5	-	-
62	2013	Nigeria	mCCDA	42	Phenotypic tests	Environmental water	74	1	-	-
63	2012	New Zealand	mCCDA	42	multiplex PCR, 16S PCR Sequencing	Goat faeces	249	10	-	-
64	2007	Northern Ireland	mCCDA	37, 42	RAPD, PFGE	Cattle faeces	220	15	-	-
65	2007	Switzerland	NS	NS	AFLP, PFGE	Cattle faeces	65	1	-	Isolates characterised from a culture collection established over 6 years
66	2006	The Netherlands	Preston Broth, mCCDA	41.5	AFLP	Mouse faeces	83	1	-	Rodents caught close to pigs on organic farm.
67	2006	Bangladesh	Brucella agar + sheep blood	42	Penner serotyping	Human stool	7128	0	Diarrhoea	-
		South Africa	antibiotic-free tryptose blood agar + horse blood	37	Penner serotyping	Human stool	5635	13	Diarrhoea	-
68	2004	Germany, Italy, UK	mCCDA	42	Phenotypic tests, Multiplex PCR	Cattle faeces	Approx.. 600	11	-	-
69	2004	Finland, Norway	Preston Broth, Preston agar	37	Not specified	Reindeer faeces	2243	1	-	-
70	2002	Australia	Preston Broth, mCCDA	42	Phenotypic tests, Uniplex PCR	Ruminant meat or offal	210	13	-	Intestinal tripe for consumption tested.
71	1998	UK	Preston Broth, mCCDA	37 (4h), 42	Phenotypic tests	Sheep meat or offal	1250	10	-	-
						Sheep faeces	252	8	-	-

72	1988	Australia	FBP Broth, Preston Broth, Preston Agar	42	Phenotypic tests	Cattle faeces	120	66	-	-
						Cattle offal or meat	112	75	-	-
73	2008	South Africa	Antibiotic-free blood agar	37	Phenotypic tests	Human stool	3877	51	Diarrhoea	Summary of the original Cape Town protocol findings (Le Roux, E. and Lastovica, A. J. 1998)
74	1983	USA	Mueller-Hinton agar, 5 % defibirinated sheep blood	37	Phenotypic tests	Pig faeces	27	18	Proliferative ileitis	
							1	21	Non-proliferative ileitis enteric disease	
75	1992	Canada	CCDA	42	Phenotypic tests, PFGE	Human stool	5	5	Diarrhoea	Source of infection was likely raw milk (cattle)

REFERENCES:

1 Kim do, K. *et al.* Campylobacter hyointestinalis isolated from a human stool specimen. *Ann Lab Med* **35**, 657-659, doi:10.3343/alm.2015.35.6.657 (2015).

2 Giacomelli, M., Follador, N., Coppola, L. M., Martini, M. & Piccirillo, A. Survey of Campylobacter spp. in owned and unowned dogs and cats in Northern Italy. *Vet J* **204**, 333-337, doi:10.1016/j.tvjl.2015.03.017 (2015).

3 Samosornsuk, W. *et al.* Isolation and Characterization of Campylobacter Strains from Diarrheal Patients in Central and Suburban Bangkok, Thailand. *Jpn J Infect Dis* **68**, 209-215, doi:10.7883/yoken.JJID.2014.229 (2015).

4 Trokhymchuk, A., Waldner, C., Chaban, B., Gow, S. & Hill, J. E. Prevalence and diversity of Campylobacter species in Saskatchewan retail ground beef. *J Food Prot* **77**, 2106-2110, doi:10.4315/0362-028X.JFP-14-247 (2014).

5 Guevremont, E., Lamoureux, L., Loubier, C. B., Villeneuve, S. & Dubuc, J. Detection and characterization of Campylobacter spp. from 40 dairy cattle herds in Quebec, Canada. *Foodborne Pathog Dis* **11**, 388-394, doi:10.1089/fpd.2013.1706 (2014).

6 Platts-Mills, J. A. *et al.* Detection of Campylobacter in stool and determination of significance by culture, enzyme immunoassay, and PCR in developing countries. *J Clin Microbiol* **52**, 1074-1080, doi:10.1128/JCM.02935-13 (2014).

7 Sasaki, Y. *et al.* Prevalence and antimicrobial susceptibility of foodborne bacteria in wild boars (Sus scrofa) and wild deer (Cervus nippon) in Japan. *Foodborne Pathog Dis* **10**, 985-991, doi:10.1089/fpd.2013.1548 (2013).

8 Serraino, A. *et al.* Presence of Campylobacter and Arcobacter species in in-line milk filters of farms authorized to produce and sell raw milk and of a water buffalo dairy farm in Italy. *J Dairy Sci* **96**, 2801-2807, doi:10.3168/jds.2012-6249 (2013).

9 Mughini Gras, L. *et al.* Increased risk for Campylobacter jejuni and C. coli infection of pet origin in dog owners and evidence for genetic association between strains causing infection in humans and their pets. *Epidemiol Infect* **141**, 2526-2535, doi:10.1017/S0950268813000356 (2013).

10 Bullman, S., O'Leary, J., Corcoran, D., Sleator, R. D. & Lucey, B. Molecular-based detection of non-culturable and emerging campylobacteria in patients presenting with gastroenteritis. *Epidemiol Infect* **140**, 684-688, doi:10.1017/S0950268811000859 (2012).

11 Seo, T. H. *et al.* The origin of non-H. pylori-related positive Giemsa staining in human gastric biopsy specimens: A prospective study. *Dig Liver Dis* **43**, 23-27, doi:10.1016/j.dld.2010.04.006 (2011).

12 Oporto, B. & Hurtado, A. Emerging thermotolerant Campylobacter species in healthy ruminants and swine. *Foodborne Pathog Dis* **8**, 807-813, doi:10.1089/fpd.2010.0803 (2011).

13 Inglis, G. D., Boras, V. F. & Houde, A. Enteric campylobacteria and RNA viruses associated with healthy and diarrheic humans in the Chinook health region of southwestern Alberta, Canada. *J Clin Microbiol* **49**, 209-219, doi:10.1128/JCM.01220-10 (2011).

14 Bullman, S. *et al.* Emerging dynamics of human campylobacteriosis in Southern Ireland. *FEMS Immunol Med Microbiol* **63**, 248-253, doi:10.1111/j.1574-695X.2011.00847.x (2011).

15 Han, H. S., Lee, K. Y., Lim, S. D., Kim, W. S. & Hwang, T. S. Molecular identification of Helicobacter DNA in human gastric adenocarcinoma tissues using Helicobacter species-specific 16S rRNA PCR amplification and pyrosequencing analysis. *Oncol Lett* **1**, 555-558, doi:10.3892/ol_00000098 (2010).

16 Salihu, M. D. *et al.* Isolation and prevalence of Campylobacter species in cattle from Sokoto state, Nigeria. *Vet Ital* **45**, 501-505 (2009).

17 Hakkinen, M., Heiska, H. & Hanninen, M. L. Prevalence of Campylobacter spp. in cattle in Finland and antimicrobial susceptibilities of bovine Campylobacter jejuni strains. *Appl Environ Microbiol* **73**, 3232-3238, doi:10.1128/AEM.02579-06 (2007).

18 Neubauer, C. & Hess, M. Detection and identification of food-borne pathogens of the genera Campylobacter, Arcobacter and Helicobacter by multiplex PCR in poultry and poultry products. *J Vet Med B Infect Dis Vet Public Health* **53**, 376-381, doi:10.1111/j.1439-0450.2006.00991.x (2006).
19 Inglis, G. D. *et al.* Temporal prevalence of antimicrobial resistance in Campylobacter spp. from beef cattle in Alberta feedlots. *Appl Environ Microbiol* **72**, 4088-4095, doi:10.1128/AEM.02830-05 (2006).
20 Inglis, G. D. *et al.* Effects of subtherapeutic administration of antimicrobial agents to beef cattle on the prevalence of antimicrobial resistance in Campylobacter jejuni and Campylobacter hyointestinalis. *Appl Environ Microbiol* **71**, 3872-3881, doi:10.1128/AEM.71.7.3872-3881.2005 (2005).
21 Inglis, G. D., Kalischuk, L. D. & Busz, H. W. Chronic shedding of Campylobacter species in beef cattle. *J Appl Microbiol* **97**, 410-420, doi:10.1111/j.1365-2672.2004.02313.x (2004).
22 Inglis, G. D., Kalischuk, L. D. & Busz, H. W. A survey of Campylobacter species shed in faeces of beef cattle using polymerase chain reaction. *Can J Microbiol* **49**, 655-661, doi:10.1139/w03-087 (2003).
23 Brown, P. E. *et al.* Frequency and spatial distribution of environmental Campylobacter spp. *Appl Environ Microbiol* **70**, 6501-6511, doi:10.1128/AEM.70.11.6501-6511.2004 (2004).
24 Hanninen, M. L. *et al.* Campylobacter hyointestinalis subsp. hyointestinalis, a common Campylobacter species in reindeer. *J Appl Microbiol* **92**, 717-723 (2002).
25 Gorkiewicz, G., Feierl, G., Zechner, R. & Zechner, E. L. Transmission of Campylobacter hyointestinalis from a pig to a human. *J Clin Microbiol* **40**, 2601-2605 (2002).
26 Gebhart, C. J., Ward, G. E. & Kurtz, H. J. In vitro activities of 47 antimicrobial agents against three Campylobacter spp. from pigs. *Antimicrob Agents Chemother* **27**, 55-59 (1985).
27 Fennell, C. L. *et al.* Isolation of "Campylobacter hyointestinalis" from a human. *J Clin Microbiol* **24**, 146-148 (1986).
28 Wilson, T. M. *et al.* Porcine proliferative enteritis: serological, microbiological and pathological studies from three field epizootics. *Can J Vet Res* **50**, 217-220 (1986).
29 Edmonds, P. *et al.* Campylobacter hyointestinalis associated with human gastrointestinal disease in the United States. *J Clin Microbiol* **25**, 685-691 (1987).
30 Hill, B. D., Thomas, R. J. & Mackenzie, A. R. Campylobacter hyointestinalis-associated enteritis in Moluccan rusa deer (Cervus timorensis subsp. Moluccensis). *J Comp Pathol* **97**, 687-694 (1987).
31 Minet, J., Grosbois, B. & Megraud, F. Campylobacter hyointestinalis: an opportunistic enteropathogen? *J Clin Microbiol* **26**, 2659-2660 (1988).
32 Terzolo, H. R. Identification of campylobacters from bovine and ovine faeces. *Rev Argent Microbiol* **20**, 53-68 (1988).
33 Diker, K. S., Diker, S. & Ozlem, M. B. Bovine diarrhea associated with Campylobacter hyointestinalis. *Zentralbl Veterinarmed B* **37**, 158-160 (1990).
34 Russell, R. G., Kiehlbauch, J. A., Gebhart, C. J. & DeTolla, L. J. Uncommon Campylobacter species in infant Macaca nemestrina monkeys housed in a nursery. *J Clin Microbiol* **30**, 3024-3027 (1992).
35 Giacoboni, G. I., Itoh, K., Hirayama, K., Takahashi, E. & Mitsuoka, T. Comparison of fecal Campylobacter in calves and cattle of different ages and areas in Japan. *J Vet Med Sci* **55**, 555-559 (1993).
36 Endtz, H. P. *et al.* Genotypic diversity of Campylobacter lari isolated from mussels and oysters in The Netherlands. *Int J Food Microbiol* **34**, 79-88 (1997).
37 Atabay, H. I. & Corry, J. E. The isolation and prevalence of campylobacters from dairy cattle using a variety of methods. *J Appl Microbiol* **84**, 733-740 (1998).

38 Lawson, A. J., Shafi, M. S., Pathak, K. & Stanley, J. Detection of campylobacter in gastroenteritis: comparison of direct PCR assay of faecal samples with selective culture. *Epidemiol Infect* **121**, 547-553 (1998).
39 Busato, A., Hofer, D., Lentze, T., Gaillard, C. & Burnens, A. Prevalence and infection risks of zoonotic enteropathogenic bacteria in Swiss cow-calf farms. *Vet Microbiol* **69**, 251-263 (1999).
40 Lawson, A. J., Logan, J. M., O'Neill G, L., Desai, M. & Stanley, J. Large-scale survey of Campylobacter species in human gastroenteritis by PCR and PCR-enzyme-linked immunosorbent assay. *J Clin Microbiol* **37**, 3860-3864 (1999).
41 Misawa, N. *et al.* Isolation of Campylobacter species from zoo animals and polymerase chain reaction-based random amplified polymorphism DNA analysis. *Vet Microbiol* **71**, 59-68 (2000).
42 Hakkinen, M. & Hanninen, M. L. Shedding of Campylobacter spp. in Finnish cattle on dairy farms. *J Appl Microbiol* **107**, 898-905, doi:10.1111/j.1365-2672.2009.04269.x (2009).
43 Pezzotti, G. *et al.* Occurrence and resistance to antibiotics of Campylobacter jejuni and Campylobacter coli in animals and meat in northeastern Italy. *Int J Food Microbiol* **82**, 281-287 (2003).
44 Vandenberg, O. *et al.* Antimicrobial susceptibility of clinical isolates of non-jejuni/coli campylobacters and arcobacters from Belgium. *J Antimicrob Chemother* **57**, 908-913, doi:10.1093/jac/dkl080 (2006).
45 Nielsen, E. M. Occurrence and strain diversity of thermophilic campylobacters in cattle of different age groups in dairy herds. *Lett Appl Microbiol* **35**, 85-89 (2002).
46 Duncan, J. S., Leatherbarrow, A. J., French, N. P. & Grove-White, D. H. Temporal and farm-management-associated variation in faecal-pat prevalence of Campylobacter fetus in sheep and cattle. *Epidemiol Infect* **142**, 1196-1204, doi:10.1017/S0950268813002379 (2014).
47 de Jong, A. *et al.* Pan-European monitoring of susceptibility to human-use antimicrobial agents in enteric bacteria isolated from healthy food-producing animals. *J Antimicrob Chemother* **67**, 638-651, doi:10.1093/jac/dkr539 (2012).
48 Mshelia, G. D., Amin, J. D., Egwu, G. O., Woldehiwet, Z. & Murray, R. D. The prevalence of bovine venereal campylobacteriosis in cattle herds in the Lake Chad basin of Nigeria. *Trop Anim Health Prod* **44**, 1487-1489, doi:10.1007/s11250-012-0092-6 (2012).
49 Mahendran, V. *et al.* Prevalence of Campylobacter species in adult Crohn's disease and the preferential colonization sites of Campylobacter species in the human intestine. *PLoS One* **6**, e25417, doi:10.1371/journal.pone.0025417 (2011).
50 Kemper, N., Aschfalk, A. & Holler, C. Campylobacter spp., Enterococcus spp., Escherichia coli, Salmonella spp., Yersinia spp., and Cryptosporidium oocysts in semi-domesticated reindeer (Rangifer tarandus tarandus) in Northern Finland and Norway. *Acta Vet Scand* **48**, 7, doi:10.1186/1751-0147-48-7 (2006).
51 Jay-Russell, M. T., Bates, A., Harden, L., Miller, W. G. & Mandrell, R. E. Isolation of Campylobacter from feral swine (Sus scrofa) on the ranch associated with the 2006 Escherichia coli O157:H7 spinach outbreak investigation in California. *Zoonoses Public Health* **59**, 314-319, doi:10.1111/j.1863-2378.2012.01465.x (2012).
52 McKenna, P. *et al.* The macaque gut microbiome in health, lentiviral infection, and chronic enterocolitis. *PLoS Pathog* **4**, e20, doi:10.1371/journal.ppat.0040020 (2008).
53 Gilbert, M. J. *et al.* Occurrence, diversity, and host association of intestinal Campylobacter, Arcobacter, and Helicobacter in reptiles. *PLoS One* **9**, e101599, doi:10.1371/journal.pone.0101599 (2014).
54 Schweitzer, N. *et al.* Molecular characterization of Campylobacter lanienae strains isolated from food-producing animals. *Foodborne Pathog Dis* **8**, 615-621, doi:10.1089/fpd.2010.0754 (2011).

55 On, S. L., Atabay, H. I. & Corry, J. E. Clonality of Campylobacter sputorum bv. paraureolyticus determined by macrorestriction profiling and biotyping, and evidence for long-term persistent infection in cattle. *Epidemiol Infect* **122**, 175-182 (1999).

56 Whittier, C. A., Cranfield, M. R. & Stoskopf, M. K. Real-time PCR detection of Campylobacter spp. In free-ranging mountain gorillas (Gorilla beringei beringei). *J Wildl Dis* **46**, 791-802, doi:10.7589/0090-3558-46.3.791 (2010).

57 Liu, J. *et al.* Simultaneous detection of six diarrhea-causing bacterial pathogens with an in-house PCR-luminex assay. *J Clin Microbiol* **50**, 98-103, doi:10.1128/JCM.05416-11 (2012).

58 Menard, A., Dachet, F., Prouzet-Mauleon, V., Oleastro, M. & Megraud, F. Development of a real-time fluorescence resonance energy transfer PCR to identify the main pathogenic Campylobacter spp. *Clin Microbiol Infect* **11**, 281-287, doi:10.1111/j.1469-0691.2005.01072.x (2005).

59 Herman, L. *et al.* Routes for Campylobacter contamination of poultry meat: epidemiological study from hatchery to slaughterhouse. *Epidemiol Infect* **131**, 1169-1180 (2003).

60 Samosornsuk, W. *et al.* Isolation and characterization of <i>Campylobacter</i> strains from diarrheal patients in Bangkok and its suburb in Thailand. *Japanese Journal of Infectious Diseases* **advpub**, doi:10.7883/yoken.JJID.2014.229 (2015).

61 Gwimi, P. *et al.* Prevalence of Campylobacter species in fecal samples of pigs and humans from Zuru Kebbi State, Nigeria. *Int J One Health2015* **1**, 1-5 (2015).

62 Ugboma, A. N., Salihu, M. D., Magaji, A. A. & Abubakar, M. B. Prevalence of Campylobacter species in ground water in Sokoto, Sokoto State, Nigeria. *Vet. World* **6**, 285-287 (2013).

63 Rapp, D. & Ross, C. Prevalence of six Campylobacter species in a New Zealand dairy goat herd. *New Zealand Journal of Agricultural Research* **55**, 235-240 (2012).

64 Madden, R., Murray, K. & Gilmour, A. Carriage of four bacterial pathogens by beef cattle in Northern Ireland at time of slaughter. *Letters in applied microbiology* **44**, 115-119 (2007).

65 Keller, J., Wieland, B., Wittwer, M., Stephan, R. & Perreten, V. Distribution and genetic variability among Campylobacter spp. isolates from different animal species and humans in Switzerland. *Zoonoses and public health* **54**, 2-7 (2007).

66 Meerburg, B. G., Jacobs-Reitsma, W. F., Wagenaar, J. A. & Kijlstra, A. Presence of Salmonella and Campylobacter spp. in wild small mammals on organic farms. *Appl Environ Microbiol* **72**, 960-962, doi:10.1128/AEM.72.1.960-962.2006 (2006).

67 Alam, K. *et al.* Clinical characteristics and serotype distribution of Campylobacter jejuni and Campylobacter coli isolated from diarrhoeic patients in Dhaka, Bangladesh, and Cape Town, South Africa. *Bangladesh Journal of Microbiology* **23**, 121-124 (2006).

68 Bywater, R. *et al.* A European survey of antimicrobial susceptibility among zoonotic and commensal bacteria isolated from food-producing animals. *Journal of Antimicrobial Chemotherapy* **54**, 744-754 (2004).

69 Kemper, N., Aschfalk, A. & Höller, C. The occurrence and prevalence of potentially zoonotic enteropathogens in semi-domesticated reindeer. *Rangifer* **24**, 15-20 (2004).

70 Bensink, J. C., Dobrenov, B., Mulenga, M. P., Bensink, Z. S. & McKee, J. J. The microbiological quality of beef tripe using different processing techniques. *Meat science* **62**, 85-92 (2002).

71 Stanley, K., Wallace, J., Currie, J., Diggle, P. J. & Jones, K. Seasonal variation of thermophilic campylobacters in lambs at slaughter. *Journal of Applied Microbiology* **84**, 1111-1116 (1998).

72 Grau, F. H. Campylobacter jejuni and Campylobacter hyointestinalis in the intestinal tract and on the carcasses of calves and cattle. *Journal of Food Protection®* **51**, 857-861 (1988).

73 Lastovica, A. J. & Allos, B. M. in *Campylobacter , Third Edition* (American Society of Microbiology, 2008).

74 Gebhart, C. J., Ward, G. E., Chang, K. & Kurtz, H. J. Campylobacter hyointestinalis (new species) isolated from swine with lesions of proliferative ileitis. *Am J Vet Res* **44**, 361-367 (1983).

75 Salama, S. M., Tabor, H., Richter, M. & Taylor, D. E. Pulsed-field gel electrophoresis for epidemiologic studies of Campylobacter hyointestinalis isolates. *J Clin Microbiol* **30**, 1982-1984 (1992).

Supplementary Data 2: Table of core genes predicted across non thermophilic *Campylobacter* using wgmlst program genome profiler. functional descriptions were generated using eggnog_mapper.

Supplementary Data 3: Fasta format allele sequences for new MLST alleles.

```
>Chy_aspA_NEW_1
ATGCTTGGCGAAGACATTGACCGCGTAAGAGAAGCTAGAAACCTAGTACGCGAGATAAAC
CTAGGTGGAACAGCTATCGGTACAGGTATCAACTCTCACCCAGACTATCCAAAAGTAGTT
GAGACAAAACTTCAAGAAGTAACAAAACGTCCATTTATCACTGCTGGTAACCTTATAGAA
GCTACTCAAGACACAGGCGCTTATGTTCAAATTTCAGGTGTTTTAAAAAGAGTTAGTACA
AAACTAAGTAAAATTTGTAACGACTTAAGACTTCTAAGTAGTGGTCCAAGATGCGGACTA
AATGAGATAAACTTACCAAAAATGCAACCAGGCTCAAGCATTATGCCGGGTAAAGTAAAT
CCTGTTATCCCTGAAGTAGTAAATCAAGTTTGTTTTGCAGTTATCGGTAATGACGTAACT
GTAACTTTAGCTAGTGAAGGCGGACAACTTCAACTAAACGTATTTGAACCAGTTATT
>Chy_aspA_NEW_2
ATGCTTGGCGAAGACATCGACCGCGTAAGAGAAGCTAGAAACTTAGTACGCGAGATAAAC
CTAGGTGGAACAGCTATCGGTACAGGTATCAACTCTCACCCAGACTATCCAAAAGTAGTT
GAGACAAAACTTCAAGAAGTAACAAAACGTCCATTTATCACTGCTGGTAACCTTATAGAA
GCTACTCAAGATACAGGCGCTTATGTTCAAATTTCAGGTGTTTTAAAAAGAGTTAGTACA
AAACTAAGTAAAATTTGTAACGACTTAAGACTTCTAAGTAGTGGTCCAAGATGCGGACTA
AATGAGATAAACTTACCAAAAATGCAACCAGGCTCAAGCATTATGCCAGGTAAAGTAAAT
CCTGTTATCCCTGAAGTAGTAAATCAAGTTTGTTTTGCAGTTATCGGTAATGACGTAACT
GTAACTTTAGCTAGTGAAGGCGGACAACTTCAACTAAACGTATTTGAACCAGTTATC
>Chy_atpA__NEW_1
GCGAGCGCAAGCGACTCTGCGGCGCTTCAATATCTAGCTCCATACGCTGGTGTTACTATG
GGTGAGTATTTCCGCGATAACTCACGCCATGCACTTATCATCTATGATGATTTGAGTAAG
CATGCTGTCGCATATCGCGAAATGTCATTGATCTTAAGAAGACCGCCAGGTCGTGAAGCC
TATCCAGGTGATGTTTTCTATCTACATTCAAGACTACTTGAAAGAGCTAGCAAACTAAGC
GATAAATTAGGAGCAGGAAGTCTTACGGCTCTACCTATTATCGAGACTCAAGCAGGCGAT
GTTTCAGCTTATATTCCTACGAATGTTATTTCTATCACAGATGGTCAGATTTTCTTGGAG
AGCGACTTGTTTAACTCAGGTATCCGTCCTGCTATTAACGTTGGTCTATCTGTTAGTCGT
GTTGGCGGATCAGCTCAGATCAAAGCTATCAAGAAAGTTTCTGGAACACTTCGTCTTGAT
CTTGCTCAA
>Chy_atpA_NEW_2
GCGAGCGCAAGCGACTCTGCGGCACTTCAATATCTAGCTCCATACGCTGGTGTTACTATG
GGTGAGTATTTCCGCGATAACTCACGCCATGCACTTATCATCTATGATGATTTGAGTAAG
CATGCTGTCGCATATCGCGAAATGTCATTGATCTTAAGAAGACCGCCAGGTCGTGAAGCC
TATCCAGGTGATGTTTTCTATCTACATTCAAGACTACTTGAAAGAGCTAGCAAACTAAGC
GATAAATTAGGAGCAGGAAGTCTTACGGCTCTACCTATTATCGAGACTCAAGCAGGCGAT
GTTTCAGCTTATATTCCTACGAATGTTATTTCTATCACAGATGGTCAGATTTTCTTGGAG
AGCGACTTGTTTAACTCAGGTATCCGTCCTGCTATTAACGTTGGTCTATCTGTTAGTCGT
GTTGGCGGATCAGCTCAGATCAAAGCTATCAAGAAAGTTTCTGGAACACTTCGTCTTGAT
CTTGCTCAA
>Chy_atpA_NEW_3
GCGAGCGCAAGCGACTCTGCAGCGCTTCAATATCTAGCTCCATACGCTGGTGTTACTATG
GGTGAGTATTTCCGCGATAACTCACGCCATGCACTTATCATCTATGATGATTTGAGTAAG
CATGCTGTCGCATATCGCGAAATGTCATTGATCTTAAGAAGACCGCCAGGTCGTGAAGCC
TATCCAGGTGATGTTTTCTATCTACATTCAAGACTACTTGAAAGAGCTAGCAAACTAAGC
GATAAATTAGGAGCAGGAAGTCTTACGGCTCTACCTATTATCGAGACTCAAGCAGGCGAT
GTTTCAGCTTATATTCCTACGAATGTTATTTCTATCACAGATGGTCAGATTTTCTTGGAG
AGCGACTTGTTTAACTCAGGTATCCGTCCTGCTATTAACGTTGGTCTATCTGTTAGTCGT
GTTGGCGGATCAGCTCAGATCAAAGCTATCAAGAAAGTTTCTGGAACACTTCGTCTTGAT
CTTGCTCAA
>Chy_atpA_NEW_4
GCGAGCGCAAGCGACTCTGCGGCGCTTCAATATCTAGCTCCATACGCTGGTGTTACTATG
GGTGAGTATTTCCGCGATAACTCACGCCATGCACTTATCATCTATGATGATTTGAGTAAG
CATGCTGTCGCATATCGCGAAATGTCATTGATCTTAAGAAGACCGCCAGGTCGTGAAGCT
TATCCAGGTGATGTTTTCTATCTACATTCAAGACTACTTGAAAGAGCTAGCAAACTAAGC
GATAAATTAGGAGCAGGAAGTCTTACGGCTCTACCTATTATCGAGACTCAAGCAGGCGAT
GTTTCAGCTTATATTCCTACGAATGTTATTTCTATCACAGATGGTCAGATTTTCTTGGAG
AGCGACTTGTTTAACTCAGGTATCCGTCCTGCTATTAACGTTGGTCTATCTGTTAGTCGT
```

GTTGGTGGATCAGCTCAGATCAAAGCTATCAAGAAAGTTTCTGGAACACTTCGTCTTGAT
CTTGCTCAA
>Chy_glnA_NEW_1
GATCCATTTACTGCCGATACCACTATCATAGTAATATGCGATGTTTATGATATATATAAA
GGTGAGTTATACGAAAAATGCCCTAGAAGTATAGCTAAAAGAGCGTCACAATATCTAAGA
GATAGCGGTATAGGCGACGTTGCTTACTTTGGCCCTGAAAATGAATTCTTCGTATTTGAT
AATGTTCGTATCATCGACAAACAAAACTGCGCAATGTACGAAGTAGATACCGAAGAAGGC
GAATGGAATGACGCTAAAGAGTACAGTGATGGTTATAATACCGGTCACCGACCACGTGCA
AAAGGTGGATATTTTCCTGTTCAACCAATAGATAGTATGGTTGATCTAAGAGCTGAAATG
GTAAATGTACTTGAGCAAGTCGGACTTGAGACTTTCGTAGTTCATCATGAAGTCGCTCAA
GGACAAGGTGAGATAGGCGTCAAATTCGGTACTCTTGTAGAAGCTGCAGATAATGTT
>Chy_glnA_NEW_2
GATCCATTTACTGCCGATACCACTATCATAGTAATATGTGATGTTTATGATATCTATAAA
GGTGAGTTATACGAAAAATGCCCTAGAAGTATAGCTAAAAGAGCGATGCAATATCTAAGA
GATAGCGGTATAGGCGACGTTGCTTACTTTGGCCCTGAAAATGAATTCTTCGTATTTGAT
AATGTTCGTATCATCGACAAACAAAACTGCGCAATGTACGAAGTAGATACCGAAGAAGGC
GAATGGAATGACGCTAAAGAGTACAGTGATGGTTATAATACCGGTCACCGACCACGTGCA
AAAGGTGGATATTTTCCTGTTCAACCAATAGATAGTATGGTTGATCTAAGAGCTGAAATG
GTAAATGTACTTGAGCAAGTCGGACTTGAGACTTTCGTAGTTCATCATGAAGTCGCTCAA
GGACAAGGTGAGATAGGCGTCAAATTCGGTACTCTTGTAGAAGCTGCAGATAATGTT
>Chy_glnA_NEW_3
GATCCATTTACTGCCGATACCACTATCATAGTAATATGCGATGTTTATGATATATATAAA
GGTGAGTTATACGAAAAATGCCCTAGAAGTATAGCTAAAAGAGCGTCACAATATCTAAGA
GATAGCGGTATAGGCGACGTTGCTTACTTTGGCCCTGAAAATGAATTCTTCGTATTTGAT
AATGTTCGTATCATCGACAAACAAAACTGCGCAATGTACGAAGTAGATACCGAAGAAGGC
GAATGGAATGACGCTAAAGAGTACAGTGATGGTTATAATACCGGTCACCGCCCACGTGCA
AAAGGTGGATATTTTCCTGTTCAACCAATAGATAGTATGGTTGATCTAAGAGCTGAAATG
GTAAATGTACTTGAGCAAGTCGGACTTGAGACTTTCGTAGTTCATCATGAAGTCGCTCAA
GGACAAGGTGAGATAGGCGTCAAATTCGGTACTCTTGTAGAAGCTGCAGATAATGTT
>Chy_glnA_NEW_4
GATCCATTTACTGCCGATACCACTATCATAGTAATATGCGATGTTTATGATATATATAAA
GGTGAGTTATACGAAAAATGCCCTAGAAGTATAGCTAAAAGAGCGTCACAATATCTAAGA
GATAGCGGTATAGGCGACGTTGCTTACTTTGGCCCTGAAAATGAATTCTTCGTATTTGAT
AATGTTCGTATCATCGACAAACAAAACTGCGCAATGTACGAAGTAGATACCGAAGAAGGC
GAATGGAATGACGCTAAAGAGTACAGTGATGGTTATAATACCGGTCACCGCCCACGTGCA
AAAGGTGGATATTTTCCTGTTCAACCAGTAGATAGTATGGTTGATCTAAGAGCTGAAATG
GTAAATGTACTTGAGCAAGTCGGACTTGAGACTTTCGTAGTTCATCATGAAGTCGCTCAA
GGACAAGGTGAGATAGGCGTCAAATTCGGTACTCTTGTAGAAGCTGCAGATAATGTT
>Chy_pgm_NEW_1
GGCGGAAATATAAATTTAAATTGCGGTGCTTTACATCCTGAGAATTTGGCTAAAGAAGTT
AAACATTTAAGAGCAGATATCGGTTTTGCTTTTGATGGAGATGCCGATAGACTAGTAGTC
GTCGATGAAAACGGCAGAGTCGTTGATGGCGACGCTCTACTTGGTATTTTAGCGACTTAT
CTTGATGAAAATAAGATGCTTGATAAAAAAGAGATAGTAGCTACTGTGATGAGCAATGCT
GCGCTTGATGACTATCTAGCAAAACATAAGATCAAACTACTTCGCTCAAACGTGGGTGAT
AAATTCGTTCTTGAGATGATGAAAGAAAACGGTATAAATTTCGGTGGTGAACAGAGCGGT
CATATTATATTTAGTGATTTTTCAAAGACCGGCGACGGACTTGTTTCAGCGCTTCAAGTT
AGTGCTTGTTTGCTTGCAAAAAGCAAAAAAGCAAGTGAGATATTTGACGTTATAAAACCA
TATCCACAAAAGCTTTTAAAT
>Chy_pgm_NEW_2
GGCGGAAATATAAATTTAAATTGCGGTGCTTTACATCCTGAGAATTTGGCTAAAGAAGTT
AAACATTTAAGAGCAGATATCGGTTTTGCTTTTGATGGAGATGCTGATAGACTCGTAGTC
GTCGATGAAAATGGCAGAGTCGTTGATGGCGACGCTCTACTTGGTATTTTAGCGACTTAT
CTTGATGAAAATAAGATGCTTGATAAAAAAGAGATAGTAGCTACTGTGATGAGCAATGCT
GCGCTTGATGACTATCTAGCAAAACATAAGATCAAACTACTTCGCTCAAACGTGGGTGAT
AAATTCGTTCTTGAGATGATGAAAGAAAACGGTATAAATTTCGGTGGTGAACAGAGCGGT
CATATTATATTTAGTGATTTTTCAAAGACCGGCGACGGACTTGTTTCAGCGCTTCAAGTT
AGTGCTTGTTTGCTTGCAAAAAGCAAAAAAGCAAGTGAGATATTTGACGTTATAAAACCA
TATCCACAAAAGCTTTTAAAT

```
>Chy_tkt_NEW_1
TTATATCTTAGCGGCTATAATGTTAGCTTAGAGGATTTAAAAAACTTTAGAAAACTTGGCT
CAAAAACCCCTGGTCACCCAGAGATCGAAACTAGCGGCGTAGAGATAGCTACTGGACCTTT
AGGGCAAGGCGTTGCAAATGCAGTTGGTTTTGCTATGGCTGCAAAAAGTGCGGCAAATTTA
CTAGGTAGTGAGATCATAAATCATAAAGTTTATTGTCTTTGCGGCGATGGCGATTTAGAAG
AAGGTATCAGCTACGAGGCTTGTGCTCTTGCTGGAAAACACTGCTTAAATAACTTGGTCAT
CATATATGATAGCAACAATATCACTATAGAAGGCGATACCTCCATAGCTTGGTGTGAAGAT
GTAAAAGTACGTTTTGAGGCTGCAGGTTTTGAAGTGGCAAAGATAGACGGACACAACTTTT
ATGAGATCGAATTTGCTCTAAGCGAGGCTGCG
```
